# NADPH oxidase 2 inhibitor GSK2795039 exerts antiplatelet and antithrombotic activity

**DOI:** 10.1038/s41598-025-20250-z

**Published:** 2025-10-16

**Authors:** Eun Bee Oh, Yun Jeong Kong, Taeil Kim, Joara Jang, Hyunseong Yu, Ji Won Park, Solee Kim, Taeryeong Kim, Jisue Sohn, Tong-Shin Chang

**Affiliations:** 1 College of Pharmacy, Seoul National University, Seoul, 08826 Republic of Korea; 2 Graduate School of Pharmaceutical Sciences, Ewha Womans University, Seoul, 03760 Republic of Korea; 3 Research Institute of Pharmaceutical Sciences, Seoul National University, Seoul, 08826 Republic of Korea

**Keywords:** Platelet, ROS, NOX2 inhibitor, GSK2795039, Antiplatelet, Antithrombotic, Biochemistry, Drug discovery

## Abstract

Reactive oxygen species (ROS) production by NADPH oxidase (NOX) is a key promoter of platelet activation, making NOX inhibition an attractive antiplatelet strategy. This study evaluated the effects of the novel NOX2 inhibitor, GSK2795039, on human platelet functional responses and ROS-mediated signaling pathways. GSK2795039 effectively suppressed both extracellular and intracellular ROS production in collagen-stimulated platelets. Consequently, GSK2795039 treatment significantly inhibited collagen-induced platelet aggregation, dense-granule ATP release, and α-granule P-selectin exposure. Furthermore, GSK2795039 attenuated tyrosine phosphorylation-mediated activation of spleen tyrosine kinase (Syk), linker for the activation of T cells (LAT), vav guanine nucleotide exchange factor 1 (Vav1), and Bruton tyrosine kinase (Btk) within the collagen receptor signaling pathway, leading to decreased phospholipase Cγ2 (PLCγ2) activation and calcium mobilization. GSK2795039 also inhibited collagen-induced integrin αIIbβ3 activation, associated with increased cyclic guanosine monophosphate (cGMP) levels and vasodilator-stimulated phosphoprotein (VASP) phosphorylation. Additionally, the inhibitor reduced collagen-induced p38 mitogen-activated protein kinase (MAPK) activation, which led to lower cytosolic phospholipase A_2_ (cPLA_2_) phosphorylation and thromboxane production. GSK2795039 further decreased extracellular signal regulated kinase 5 (ERK5) activation, thereby limiting procoagulant phosphatidylserine exposure. Notably, thrombus formation induced by platelet adhesion to collagen was abolished in the presence of GSK2795039. In vivo, GSK2795039 administration markedly inhibited arterial thrombosis. This is the first study to demonstrate that GSK2795039 suppresses collagen-induced ROS production, platelet activation, and thrombus formation, highlighting its potential as a therapeutic agent for thrombotic and cardiovascular diseases.

**Supplementary Information:**

The online version contains supplementary material available at 10.1038/s41598-025-20250-z.

## Introduction

Platelets are essential cells involved in hemostasis and thrombosis, continually monitoring the vascular system to quickly detect damaged or inflamed sites. Upon vascular injury, platelets adhere to the subendothelium by binding von Willebrand factor and glycoprotein (GP) Ib receptors, leading to their firm attachment to subendothelial collagen via collagen receptor GPVI. This interaction initiates a signaling cascade that promotes platelet activation, secretion, and aggregation^1,2^. Through these processes, platelets function to seal blood vessel injuries, preventing blood loss; however, in certain pathological conditions, uncontrolled platelet activation can lead to thrombus formation. Thrombotic disorders pose significant health risks globally and are major causes of mortality and disability, making effective prevention and treatment strategies essential. Currently, antiplatelet therapies play a central role in reducing the risk of thrombotic events for patients with cardiovascular, cerebrovascular, and peripheral artery disease.

NADPH oxidase (NOX) enzymes, as primary sources of reactive oxygen species (ROS), are pivotal in both physiological and pathological platelet signaling^3^. A substantial body of research has established the importance of NOX-derived ROS in platelet function and signaling^4–7^. Furthermore, NOX activity appears to increase platelet sensitivity, contributing to the heightened responsiveness seen in thrombotic conditions^5–8^. The NOX family comprises seven members-NOX1–5 and the dual oxidases Duox1 and Duox2. Among these, human platelets express NOX1, 2, and 4, with NOX1 and NOX2 playing particularly crucial roles in modulating platelet function, as demonstrated in studies using triple NOX knockout mice^6,7^. NOX2, in particular, is central to ROS production and platelet activation, as evidenced by various experimental and clinical studies^7,9–11^. In NOX2-deficient mouse models, platelets exhibit reduced thrombotic activity and platelet-leukocyte interactions, highlighting NOX2’s role in thrombosis and inflammation^7,12^. The importance of NOX2 in human platelets is further underscored by observations that platelets from individuals with X-linked chronic granulomatous disease produce less ROS and exhibit lower the cluster of differentiation 40 (CD40) ligand expression upon activation^7,12^. Interestingly, despite NOX2’s contribution to thrombotic processes, patients with this condition^11^ and NOX2-deficient mice^7^ do not show increased bleeding, indicating that NOX2’s role may be more specific to thrombosis than hemostasis. Accordingly, NOX2 has emerged as a promising therapeutic target to inhibit platelet function while minimizing bleeding risk.

GSK2795039, a novel NOX2-specific inhibitor, has been shown to inhibit NOX2-derived ROS production in an inflammation model^13^. Although GSK2795039 is known to inhibit collagen-induced platelet aggregation^14^, its precise mechanisms in modulating NOX2-mediated ROS pathways that contribute to platelet activation and thrombus formation remain unexplored. This study aims to assess whether GSK2795039 can effectively inhibit platelet activation and thrombus formation by targeting ROS production and interrupting subsequent ROS-mediated signaling pathways.

## Results

### GSK2795039 attenuates collagen-stimulated platelet aggregation and ROS production

**Fig. 1:**
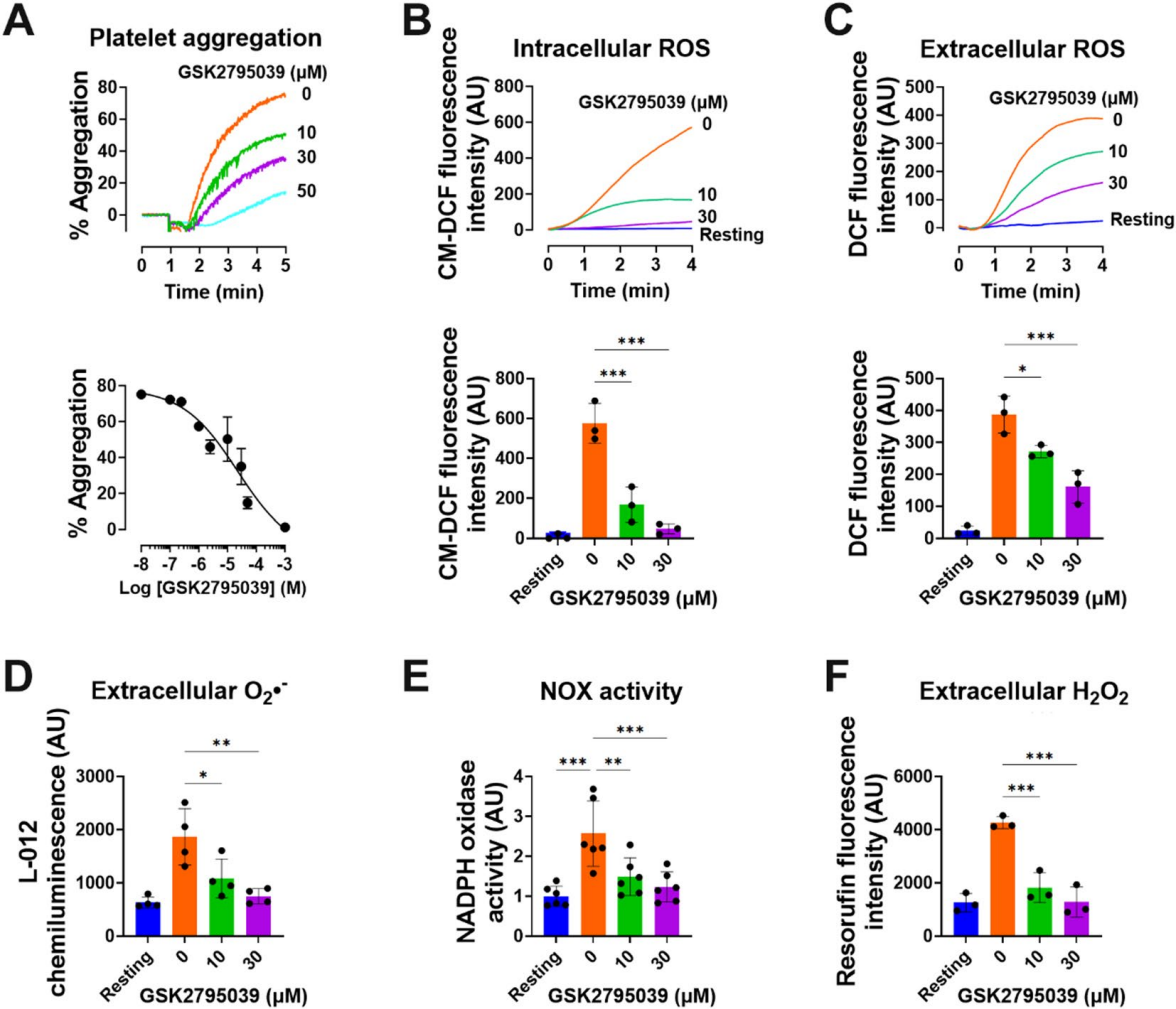
GSK2795039 attenuates collagen-stimulated platelet aggregation and ROS production. (**A**) Washed human platelets (5 × 10^8^/mL) were pretreated with vehicle (0.5% DMSO) or various concentrations of GSK2795039 for 5 min before stimulation with collagen (10 µg/mL). Platelet aggregation was monitored by measuring changes in light transmission, with 100% transmission defined as the buffer control. Data represent the mean ± standard deviation from three independent donors. (**B**–**D**) For analyzing the levels of intracellular ROS (**B**), extracellular ROS (**C**), and extracellular superoxide anion (**D**), following CM-H_2_DCFDA loading (**B**); in the presence of DCFH_2_ (**C**) or L-012 (**D**), washed human platelets were treated as in A. (**B**,** C**) Fluorescence change was measured in a fluorometer cuvette under constant stirring conditions. Upper panels show representative traces. (**D**) Chemiluminescence was measured in a luminometer. (**E**) NOX activity assay. Washed human platelets were treated as in A. Cell lysates were prepared and then incubated with NADPH. Superoxide anion production in the lysates was measured by a chemiluminescence assay using L-012. (**F**) To quantify extracellular H_2_O_2_, the supernatant from the reaction mixture was incubated with Amplex Red reagent, and resorufin fluorescence was measured after 30 min. AU, arbitrary units. All data represent the mean ± standard deviation. Statistical significance: * *p <* 0.05, ** *p <* 0.01 and *** *p <* 0.001.

Collagen receptor GPVI is essential for platelet adhesion, activation, and accumulation^1^. As NOX2 promotes GPVI-stimulated platelet activation^7^, we investigated the impact of NOX2 inhibitor GSK2795039 on human platelet aggregation after collagen stimulation. GSK2795039 significantly suppressed collagen-induced aggregation in a concentration-dependent manner, with an IC_50_ value of 22.6 µM (Fig. 1A). To confirm whether this inhibitory effect also occurs in a physiological plasma environment, we evaluated collagen-induced platelet aggregation using platelet-rich plasma (PRP). As shown in Fig. S1, preincubation of PRP with GSK2795039 markedly attenuated collagen-induced platelet aggregation in a dose-dependent manner, consistent with results obtained using washed platelets.

We investigated the effect of GSK2795039 on thrombin- or U46619-induced platelet aggregation, as NOX2-deficient mouse platelets showed partial inhibition^7^ and GSK2795039 had no inhibitory effect on human platelets^14^. Despite inhibiting collagen-induced platelet aggregation, GSK2795039 did not influence thrombin- or U46619-induced aggregation at 50 µM concentration (Supplementary Fig. S2).

Next, we examined the effect of GSK2795039 on collagen-induced intracellular ROS levels by evaluating intraplatelet redox status using 5-(and 6)-chloromethyl-2′,7′-dichlorodihydrofluorescein diacetate (CM-H_2_DCFDA), which is sensitive to oxidation and can detect a wide range of ROS, including peroxides and hydroxyl radicals (Fig. 1B). Similar to earlier research^15–17^, collagen stimulation significantly increased ROS generation in human platelets. GSK2795039 concentration-dependently reduced collagen-induced intracellular ROS. We examined the impact of GSK2795039 on collagen-induced extracellular ROS generation using 2′,7′-dichlorodihydrofluorescein (DCFH_2_) a redox-sensitive and membrane-impermeable probe^18^. Collagen-stimulated platelets significantly increased extracellular ROS production, supporting prior findings^15,16^. GSK2795039 concentration-dependently reduced collagen-induced extracellular ROS increase (Fig. 1C).

To directly evaluate whether the inhibitory effect of GSK2795039 on ROS generation is attributable to NOX2 suppression, we first quantified extracellular superoxide anion levels in intact platelets using L-012-based real-time chemiluminescence. Collagen stimulation induced a marked increase in superoxide release compared to resting conditions, which was significantly attenuated by GSK2795039 in a concentration-dependent manner (Fig. 1D). Next, to assess enzymatic NOX activity more directly, platelet lysates were incubated with NADPH and L-012 to monitor NADPH oxidase–driven superoxide production. Collagen stimulation markedly enhanced NOX activity relative to resting platelets, whereas pretreatment with GSK2795039 resulted in a dose-dependent suppression of NOX enzymatic activity (Fig. 1E). These findings confirm that GSK2795039 diminishes collagen-induced ROS generation by directly inhibiting NOX2-dependent superoxide production.

The NOX complex generates extracellular superoxide, which spontaneously dismutates to H_2_O_2_^19^. Anionic superoxide is not membrane permeable, whereas uncharged H_2_O_2_ may diffuse into the cytosol, increasing intracellular ROS. Using the H_2_O_2_-specific probe Amplex Red, we found that GSK2795039 significantly inhibits the collagen-induced increase of extraplatelet H_2_O_2_ (Fig. 1F), as extraplatelet superoxide does not affect platelet aggregation^5^. These results suggest that GSK2795039 reduces collagen-stimulated platelet activation by decreasing ROS production.

### GSK2795039 inhibits protein tyrosine phosphorylation in collagen-stimulated platelets

**Fig. 2:**
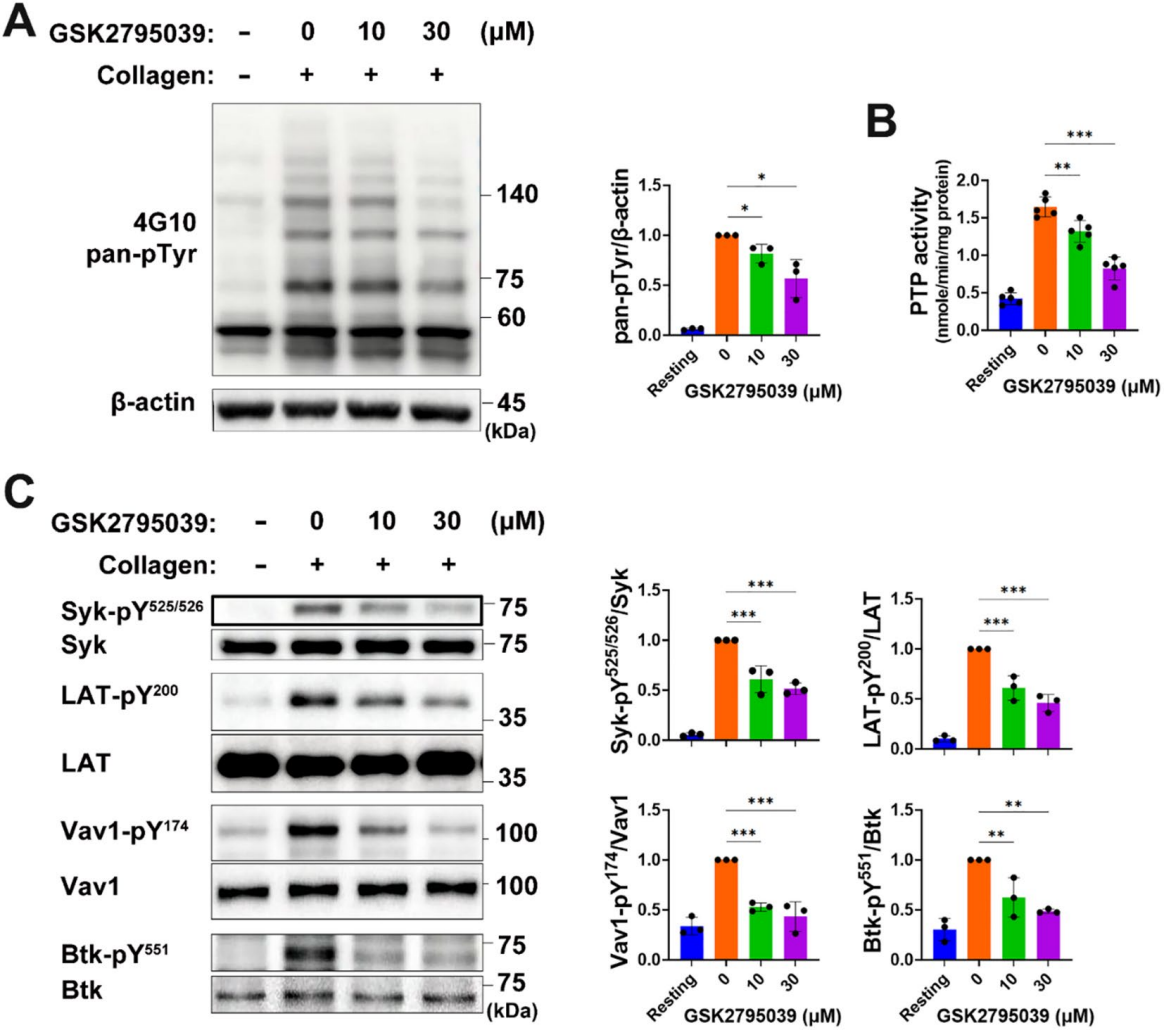
GSK2795039 inhibits protein tyrosine phosphorylation in collagen-stimulated platelets. Washed platelets were treated with vehicle or GSK2795039 at the indicated concentrations for 5 min and stimulated with collagen (10 µg/mL) for 2 min. (**A**) Total protein tyrosine phosphorylation levels were analyzed by immunoblotting, with β-actin serving as a loading control. (**B**) After treatment with iodoacetamide and N-ethylmaleimide in a hypoxic chamber, platelet lysates were further incubated with dithiotreitol. The activity of total protein tyrosine phosphatases (PTPs), which were recovered from oxidation, was determined using tyrosine phosphopeptide as the substrate. (**C**) The phosphorylation of Syk, LAT, Vav1, and Btk at specific tyrosine residues was assessed by immunoblotting with phospho-specific antibodies. Representative immunoblots are shown alongside quantitative analysis of band intensity. Data represent the mean ± standard deviation. Statistical significance: * *p <* 0.05, ** *p <* 0.01 and *** *p <* 0.001.

Protein-tyrosine phosphatases (PTPs) are susceptible to ROS-induced oxidative inactivation due to reactive cysteine thiolate in the catalytic region, leading to increased phosphorylation^20^. GPVI-stimulated platelet signaling involves protein tyrosine phosphorylation cascades^21^, making ROS-mediated PTP oxidation and deactivation potentially beneficial^17,22,23^. GSK2795039 reduced ROS rise in collagen-stimulated platelets, thus we evaluated its impact on total protein tyrosine phosphorylation. Figure 2A indicates that collagen-induced protein tyrosine phosphorylation of numerous platelet proteins increased considerably from baseline. In response to collagen stimulation, platelets pretreated with GSK2795039 reduced protein tyrosine phosphorylation.

To explore whether the reduced protein tyrosine phosphorylation was due to preserved PTP activity, we directly assessed the enzymatic function of PTPs following GSK2795039 treatment. Because PTPs can undergo reversible oxidative inactivation by ROS, platelet lysates were pretreated with cysteine-specific alkylating agents under anoxic conditions to prevent further oxidation and to stabilize the thiol redox state. Subsequent reduction of reversibly oxidized cysteines allowed quantification of the active pool of PTPs. As shown in Fig. 2B, collagen stimulation significantly decreased PTP activity, whereas GSK2795039 treatment restored PTP enzymatic activity in a concentration-dependent manner. These findings support the notion that GSK2795039 prevents ROS-dependent PTP oxidation, thereby preserving phosphatase activity and counteracting aberrant phosphorylation during GPVI signal propagation.

We examined the tyrosine phosphorylation-based activation of spleen tyrosine kinase (Syk), linker for the activation of T cells (LAT), vav guanine nucleotide exchange factor 1 (Vav1), and Bruton tyrosine kinase (Btk) to determine whether GSK2795039 affects GPVI signaling cascade activation. In collagen-stimulated platelets, autophosphorylation of Syk-Tyr^525^/Tyr^526^ on activation loops implies kinase activity^24,25^. Phosphorylation of LAT tyrosine residues like Tyr^200^ by Syk enables src homology 2 (SH2) domain-containing proteins to attach and form a signaling complex^21,26,27^. Phosphorylations of Vav1-Tyr^174^ and Btk-Tyr^551^ in this complex cause phospholipase Cγ2 (PLCγ2)-Tyr^753^ phosphorylation contributing to its activation^28–30^. Western blot examination with phospho-specific antibodies showed that collagen stimulation increased the phosphorylation of Tyr^525^/Tyr^526^ on Syk, Tyr^200^ on LAT, Tyr^174^ on Vav1, and Tyr^551^ on Btk (Fig. 2C). The tyrosine phosphorylation-based activation of Syk, LAT, Vav1, and Btk in collagen-stimulated platelets was distinctively inhibited by GSK2795039 treatment. Taking into account that phosphatases like low molecular weight-PTP (LMW-PTP), src homology 2-containing inositol phosphatase 1 (SHIP-1), phosphatase and tensin homolog deleted on chromosome 10 (PTEN), and src homology 2-containing PTP2 (SHP-2) can lower GPVI-stimulated platelet activation by dephosphorylating many substrates^17,31–33^, our results show that GSK2795039 protects PTPs from oxidative inactivation by blocking NOX2-mediated ROS production, which lowers GPVI signaling in platelets.

### GSK2795039 suppresses PLCγ2/PKC/Ca^2+^ pathway in collagen-stimulated platelets

**Fig. 3:**
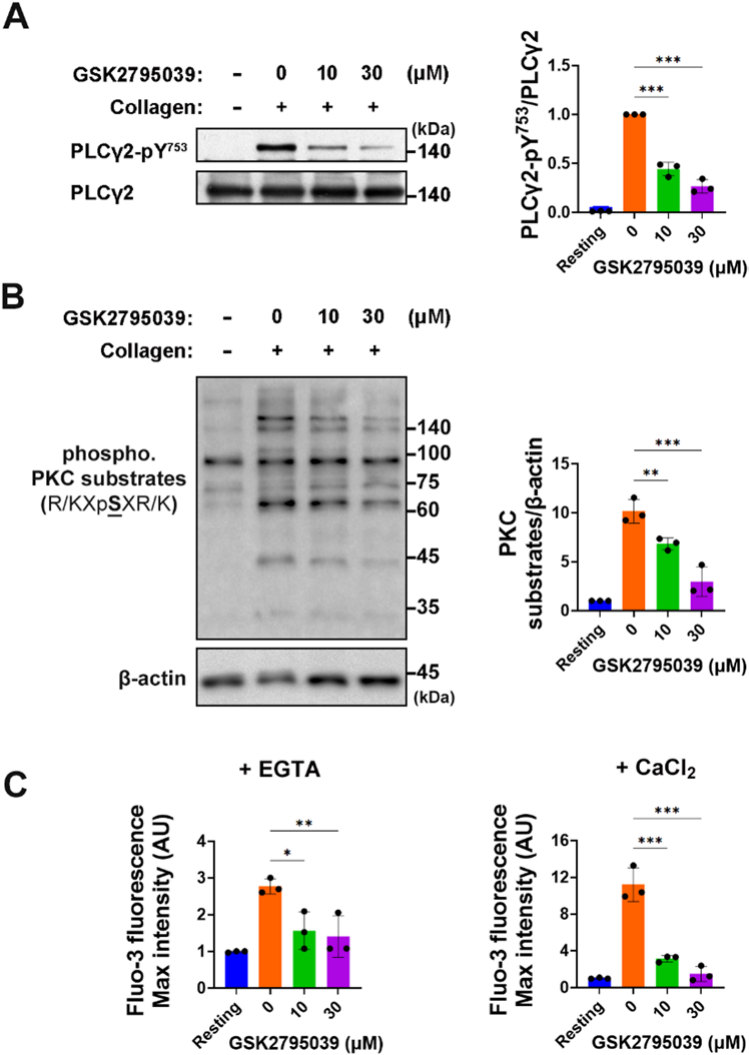
GSK2795039 suppresses PLCγ2/PKC/Ca^2+^ pathway in collagen-stimulated platelets. (A and B) Washed human platelets were treated with vehicle or GSK2795039 for 5 min and stimulated with collagen (10 µg/mL) for 2 min. Phosphorylation of PLCγ2 at Tyr^753^ (**A**) and PKC substrates (**B**) were analyzed by immunoblotting, respectively. Representative immunoblots are shown alongside quantitative analysis of band intensity. (**C**) Intracellular calcium mobilization was measured in Fluo-3-AM-loaded washed human platelets stimulated with collagen for 2 min in the presence of either 0.5 mM EGTA (left) or 1 mM extracellular CaCl_2_ (right). Fluorescence intensity expressed in arbitrary units (AU) was recorded, and maximum intensity values are presented. All data represent the mean ± standard deviation. Statistical significance: * *p <* 0.05, ** *p <* 0.01 and *** *p <* 0.001.

The activation of Vav1 and Btk through tyrosine phosphorylation is associated with the phosphorylation of Tyr^753^ on the downstream target PLCγ2 following collagen stimulation, thereby enhancing its activity^28–30^. By inhibiting upstream molecules, GSK2795039 allowed us to study PLCγ2 phosphorylation at Tyr^753^ in collagen-stimulated platelets. Figure 3A demonstrates that GSK2795039 inhibits collagen-induced PLCγ2 phosphorylation at Tyr^753^. In collagen-stimulated platelets, PLCγ2-mediated diacylglycerol and inositol-1,4,5-trisphosphate (IP_3_) production activates protein kinase C (PKC) and increases cytosolic Ca^2+^ by releasing Ca^2+^ from intracellular stores^21,34^. To determine whether GSK2795039 affects PKC signaling, we examined the phosphorylation status of classical PKC substrate motifs^35^ by immunoblotting. Upon collagen stimulation, a characteristic set of PKC-dependent phosphoprotein bands was observed, indicating pathway activation. Pretreatment with GSK2795039 markedly attenuated these phosphorylation patterns, suggesting inhibition of PKC activation (Fig. 3B).

**Fig. 4:**
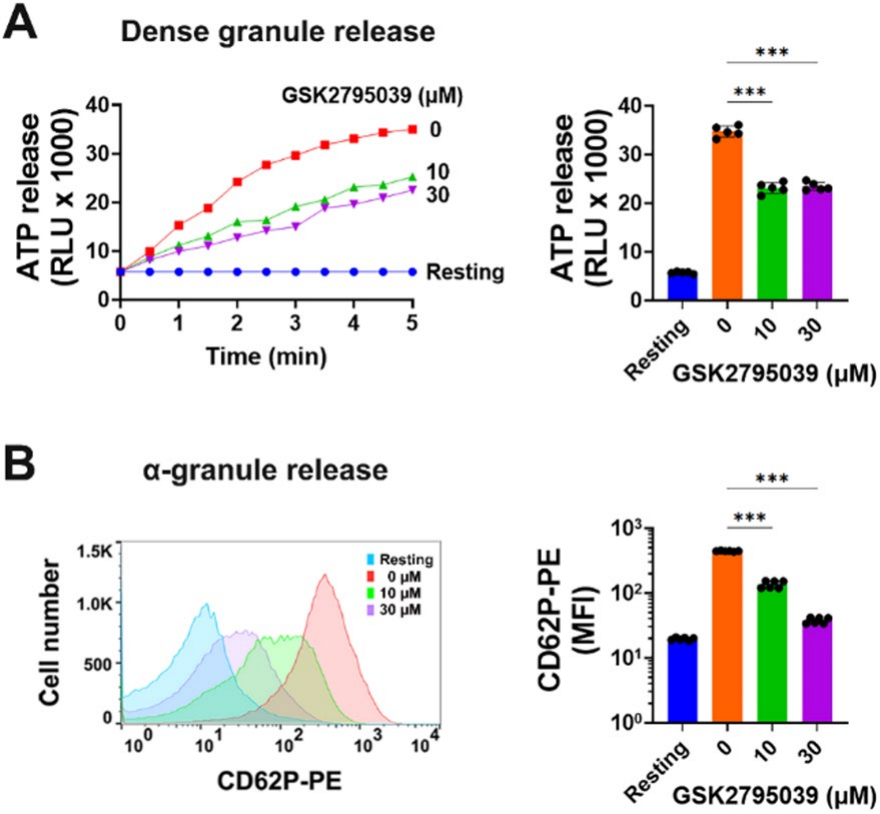
GSK2795039 inhibits collagen-stimulated granule release in platelets. Washed human platelets were treated with vehicle or GSK2795039 for 5 min and stimulated with collagen (10 µg/mL) for 5 min. (**A**) Platelet dense granule secretion was assessed by measuring ATP release using a luciferin-luciferase assay. Data are presented as representative tracings and maximum luminescence (relative luminescence units, RLU) generated by ATP. (B) Surface expression of P-selectin (CD62P) as a marker of α-granule secretion was measured by flow cytometry after staining with CD62P-PE. Representative histograms and mean fluorescence intensity (MFI) values are shown. All data represent the mean ± standard deviation, with statistical significance indicated: ***p < 0.001.

The inhibition of PKC signaling by GSK2795039 carries significant mechanistic implications, given PKC’s established role in promoting the phosphorylation of p47 phagocyte oxidase (p47phox) at Ser^304^—an essential event for NOX2 activation^36^. Our data indicate that GSK2795039 attenuates p47phox phosphorylation (Supplementary Fig. S3), potentially through disruption of the PLCγ2–diacylglycerol–PKC axis (Fig. 4A and B), thereby hindering the assembly of the active NOX2 complex. In addition, consistent with a previous report^13^, GSK2795039 likely interferes with the NADPH-binding site of gp91phox, effectively blocking electron transfer and subsequent ROS generation. These findings suggest that GSK2795039 exerts dual inhibitory actions by impeding both the catalytic function and upstream regulatory mechanisms of NOX2, ultimately reducing the membrane recruitment of cytosolic NOX components.

We used Fluo-3-AM to measure cytosolic Ca^2+^. When triggered without external Ca^2+^, platelets release Ca^2+^ from internal reserves, increasing cytosolic Ca^2+^. We measured cytosolic Ca^2+^ in 0.5 mM EGTA (Fig. 3B). GSK2795039 effectively decreased collagen-induced Ca^2+^ mobilization from internal reserves. GSK2795039 reduced collagen-induced cytosolic Ca^2+^ rise even in the presence of external Ca^2+^ (Fig. 3C). These data show that GSK2795039 inhibits Ca^2+^ mobilization from internal and external sources.

### GSK2795039 inhibits collagen-stimulated granule release in platelets

Release of active chemicals from dense and α-granules after activation promotes additional platelet activation. PLCγ2-mediated PKC activation and increased cytosolic Ca^2+^ promote granule release in collagen-stimulated platelets^34,37^. GSK2795039 inhibited PLCγ2, thus we investigated its influence on collagen-induced platelet dense-granule release. Results showed considerable inhibition of ATP release from platelets (Fig. 4A). Platelets and leukocytes bind together via P-selectin. Active platelets exhibit it following α-granule degranulation^38^. Using PE-labelled anti-P-selectin antibody (CD62P-PE), flow cytometry demonstrated that collagen increases platelet surface P-selectin, which GSK2795039 greatly attenuates (Fig. 4B).

### GSK2795039 suppresses the p38 MAPK/cPLA_2_/TXA_2_ signaling pathway in collagen-stimulated platelets

**Fig. 5:**
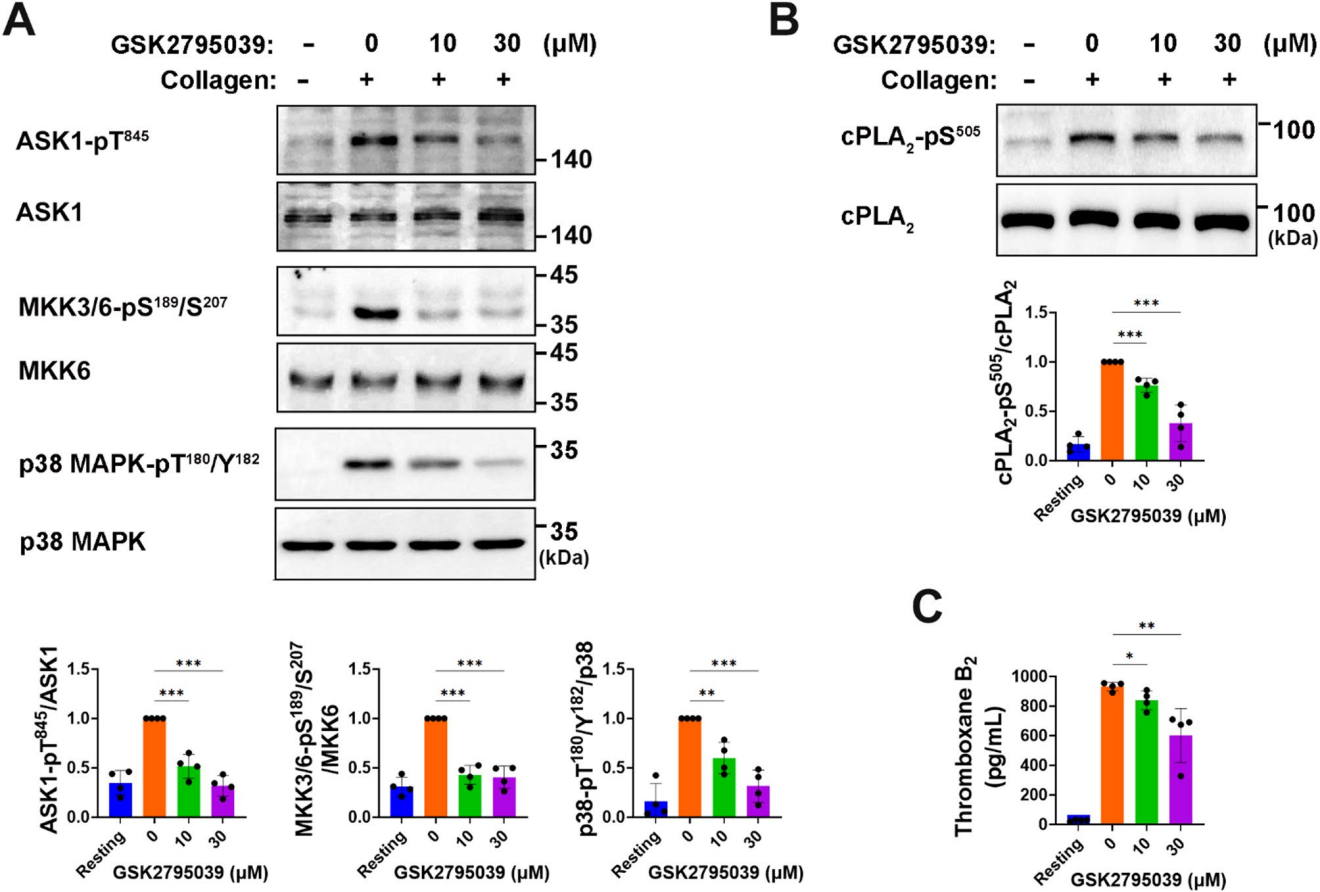
GSK2795039 suppresses the p38 MAPK/cPLA_2_/TXA_2_ signaling pathway in collagen-stimulated platelets. (**A**,** B**) Washed human platelets were pretreated with vehicle or GSK2795039 and stimulated with collagen (10 µg/mL) for 2 min. Phosphorylation of ASK1, MKK3/6, p38 MAPK (**A**), and cPLA_2_ (**B**) was analyzed by immunoblotting. Representative immunoblots and quantitative data are presented. (**C**) Thromboxane B_2_ (TXB_2_), a stable metabolite of TXA_2_, was measured in the supernatant of collagen-stimulated platelets using an ELISA. All data represent the mean ± standard deviation. Statistical significance: *p < 0.05, **p < 0.01, ***p < 0.001.

Thromboxane A_2_ (TXA_2_) is a secondary-wave agonist that enhances collagen-induced platelet aggregation^39^. Platelets activated by collagen need cytosolic phospholipase A_2_ (cPLA_2_) to create TXA_2_ by releasing arachidonic acid from the plasma membrane^40^. In collagen-stimulated human platelets, p38 mitogen-activated protein kinase (MAPK) phosphorylates cPLA_2_ at Ser^505^, resulting in TXA_2_ synthesis^41^. Studies indicate that the absence of p47phox, a regulatory subunit of NOX2, or the selective inhibition of NOX2 hinders the activation of p38 MAPK in GPVI-stimulated platelets^9,42^. Apoptosis signal-regulating kinase 1 (ASK1), a MAPK kinase kinase family member, regulates p38 MAPK/cPLA_2_/TXA_2_ synthesis in mouse platelets upon agonist activation^43^. In human platelets, H_2_O_2_ activates ASK1, phosphorylating MAPK kinases (MKK) 3, 4, and 6, which in turn activating p38 MAPK^44^. Additionally, removing ROS reduces collagen-induced TXA_2_ synthesis in human platelets^4^. Thus, we investigated if GSK2795039 influences ASK1/p38 MAPK signaling axis to activate cPLA_2_. Collagen stimulation of platelets led to the activation of MKK3/6, which was associated with the phosphorylation of ASK1 at Thr^838^ (a marker of kinase activity) and p38 MAPK at Thr^180^/Tyr^182^ (reflecting activation) (Fig. 5A). This was followed by an increase in cPLA_2_ phosphorylation at Ser^510^ (Fig. 5B). Preincubation with GSK2795039 greatly decreased these phosphorylations (Fig. 5A and B). We next examined its effects on the generation of TXB_2_, a stable TXA_2_ metabolite. In collagen-stimulated platelets, GSK2795039 reduced TXB_2_ synthesis (Fig. 5C). These data suggest that the antiplatelet effect of GSK2795039 is linked to the downregulation of the ROS-driven ASK1/p38 MAPK/cPLA_2_/TXA_2_ signaling cascade.

### GSK2795039 reduces ERK5 activation and PS exposure in collagen-stimulated platelets

**Fig. 6:**
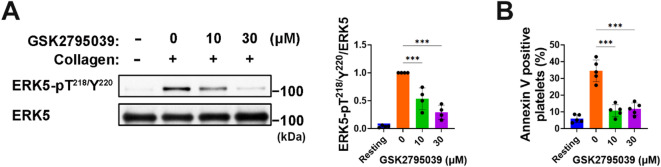
GSK2795039 reduces ERK5 activation and PS exposure in collagen-stimulated platelets. (**A**) Washed human platelets were treated with GSK2795039 or vehicle and stimulated with collagen (10 µg/mL) for 2 min. Phosphorylation of ERK5 at Thr^218^/Tyr^220^ was analyzed by immunoblotting. Representative immunoblots and quantitative data are presented. (**B**) Washed human platelets surface exposure of phosphatidylserine was assessed by annexin V-FITC staining and analyzed using flow cytometry. All data represent the mean ± standard deviation. Statistical significance: ***p < 0.001.

NOX-derived ROS in stimulated platelets activate extracellular signal regulated kinase 5 (ERK5), a redox-sensitive MAP kinase^45,46^. ROS-activated ERK5 stimulates caspases, boosting procoagulant phosphatidylserine (PS) externalization and fibrin production while the mechanism is uncertain^46^. Thus, we investigated whether GSK2795039 influences ERK5’s extracellular PS exposure enhancement. GSK2795039 preincubation inhibited platelet collagen-induced ERK5 phosphorylation at Thr^218^/Tyr^220^ (Fig. 6A). Next, using flow cytometry, we examined GSK2795039’s impact on platelet procoagulant reactions by assessing PS exposure marker annexin V-FITC binding to platelets. PS exposure increased after collagen stimulation, whereas GSK2795039 pretreatment decreased annexin V-positive platelets (Fig. 6B). GSK2795039’s capacity to reduce ROS generation may prevent procoagulant states in cardiovascular disease by reducing ROS-sensitive ERK5 activation.

### GSK2795039 modulates the cGMP/PKG/VASP/integrin-αIIbβ3 signaling pathway in collagen-stimulated platelets

**Fig. 7:**
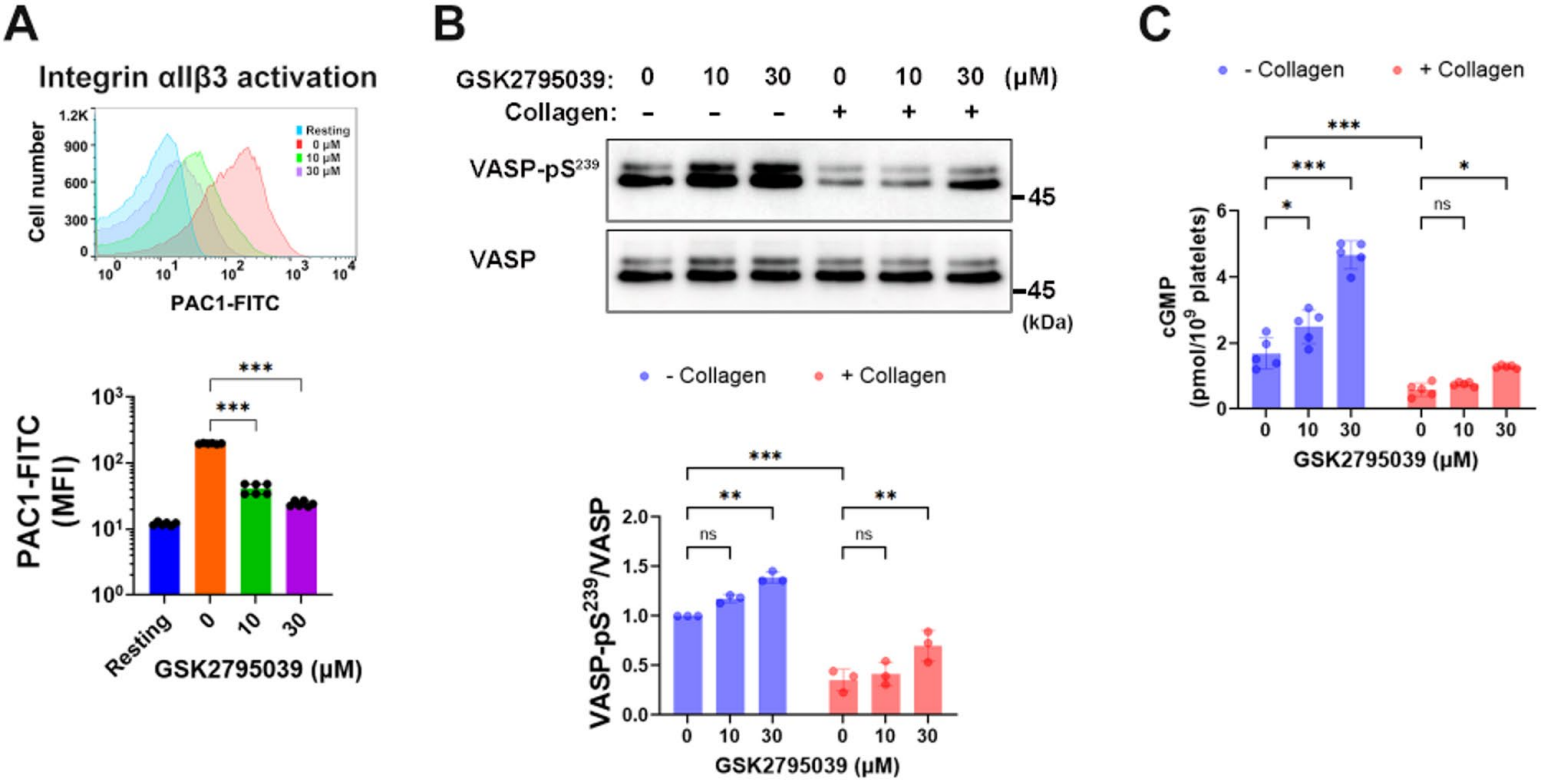
GSK2795039 modulates the cGMP/PKG/VASP/integrin-αIIbβ3 signaling pathway in collagen-stimulated platelets. Washed human platelets were treated with GSK2795039 (10 or 30 µM) or vehicle for 5 min, then stimulated with collagen (10 µg/mL). (**A**) Activation of integrin-αIIbβ3 was assessed by PAC1-FITC binding using flow cytometry after 5 min of collagen stimulation. Representative histograms and mean fluorescence intensity (MFI) are shown. (**B**) Phosphorylation of VASP at Ser^239^ was analyzed by immunoblotting after 2 min of collagen stimulation. Representative blots and densitometric quantification are presented. (**C**) Intraplatelet cGMP levels were quantified by ELISA after 2 min of collagen stimulation. All data represent the mean ± standard deviation. Statistical significance: *p < 0.05, **p < 0.01, ***p < 0.001 and *ns*, *p* > 0.05 (not significant).

Activation of integrin-αIIbβ3 leads to high-affinity fibrinogen binding, affecting platelet adhesion and aggregation^47^. NOX-derived ROS are essential for activating integrin-αIIbβ3 in GPVI-stimulated platelets^6^. Therefore, we examined the impact of GSK2795039 on this signaling pathway. The FITC-labelled anti-active-integrin αIIbβ3 antibody (PAC1-FITC), which identifies the activated conformation of αIIbβ3, showed that GSK2795039 reduced the rise in active conformational changes of integrin-αIIbβ3 in response to collagen stimulation (Fig. 7A).

The adaptor molecule vasodilator-stimulated phosphoprotein (VASP) inhibits platelet activation by regulating integrin-αIIbβ3 activation^48,49^ and links cyclic nucleotide-dependent pathways to actin remodelling, regulating cytoskeletal dynamics^50^. The function of VASP in cyclic guanosine monophosphate (cGMP)-mediated suppression of platelet integrin-αIIbβ3 activation and aggregation has been demonstrated in VASP-null mouse platelets, albeit the mechanism is unclear^48,49^. VASP phosphorylation at Ser^239^ inhibits platelet activation via the cGMP/protein kinase G (PKG) signaling pathway, negatively affecting platelet responsiveness^49,51^. In collagen-stimulated conditions, triple NOX-deficient mouse platelets showed greater VASP Ser^239^ phosphorylation compared to the wild type, accompanied by higher intraplatelet cGMP levels in resting conditions^6^. Research indicates that NOX2-derived ROS in platelets block the cGMP/PKG signaling pathway^8^.

To examine the effect of GSK2795039 on regulating platelet cGMP signaling, we first examined whether GSK2795039 alone could affect VASP phosphorylation under resting conditions. Treatment of platelets with GSK2795039 significantly increased phosphorylation of VASP at Ser^239^ in a dose-dependent manner (Fig. 7B, blue bars), accompanied by elevated intracellular cGMP levels (Fig. 7C, blue bars), indicating activation of the NO/cGMP/PKG pathway. The mechanism was confirmed to be PKG-dependent, as the effect was abolished by PKG inhibitor Rp-8-pCPT-cGMPS but not affected by PKA inhibitor H-89 (Supplementary Fig. S4A).

As shown in Fig. 7B and C, collagen stimulation decreased both pVASP and cGMP, and these reductions were significantly reversed by GSK2795039 pretreatment. Supplementary Fig. S4B demonstrates that ROS scavengers Mn(III)TMPyP and Tempol similarly rescued VASP phosphorylation. These findings support a model in which collagen-induced ROS generation decreases NO bioavailability or inhibits soluble guanylate cyclase (sGC), resulting in reduced cGMP synthesis and PKG activation. Consistently, treatment with either an NO donor (DEA-NONOate) or a cGMP analog (8-pCPT-cGMP) in combination with GSK2795039 further augmented pVASP levels (Supplementary Fig. S4C and D), reinforcing the conclusion that ROS suppresses the NO/cGMP/PKG pathway in collagen-stimulated platelets.

Finally, inhibition of PKG by Rp-8-pCPT-cGMPS reversed the anti-aggregatory effect of GSK2795039, whereas PKA inhibition by H-89 had no such effect and instead enhanced aggregation inhibition (Supplementary Fig. S4E). Together, these results show that the modulation of the ROS-mediated cGMP/PKG/VASP/integrin-αIIbβ3 signaling pathway by GSK2795039 contributes to its inhibitory effect on collagen-induced platelet aggregation.

### GSK2795039 inhibits thrombus formation under flow and reduces thrombosis in vivo without impairing hemostasis

**Fig. 8:**
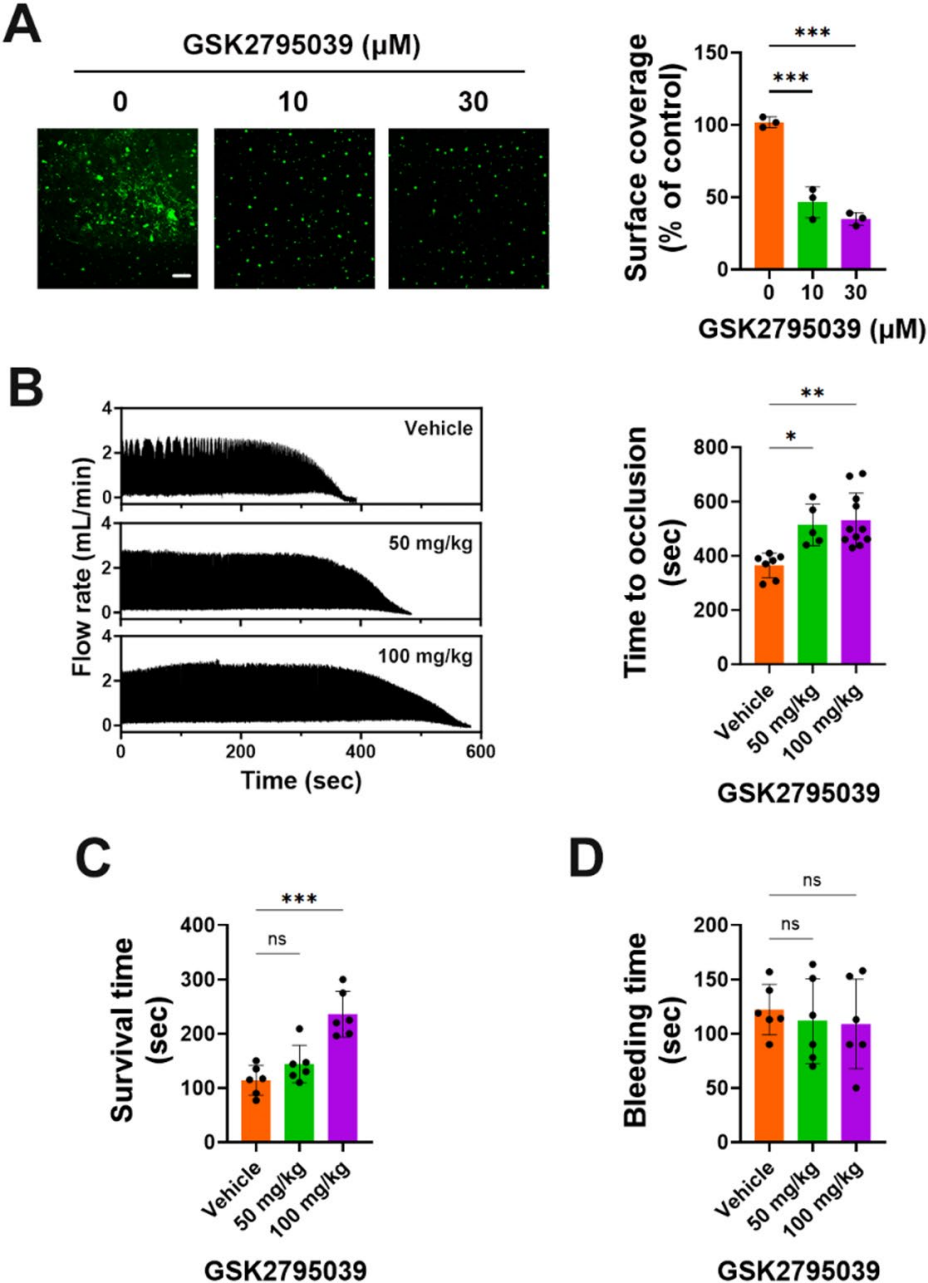
GSK2795039 inhibits thrombus formation under flow and reduces thrombosis in vivo without impairing hemostasis. (**A**) Washed human platelets labeled with DiOC6 were perfused over a collagen-coated surface at a shear rate, and platelet adhesion and thrombus formation were visualized. Representative images and quantification of surface coverage are presented. Scale bar represents 100 μm. (**B**–**D**) Mice were pretreated with vehicle or GSK2795039 (50 or 100 mg/kg). (**B**) Mice were subjected to ferric chloride-induced carotid artery injury. Occlusion times were recorded using a flowmeter. The left panel shows representative tracings of blood flow. (**C**) Pulmonary thromboembolism was induced by retro-orbital injection of collagen and epinephrine. Time for thromboembolic death was measured and plotted. (**D**) Bleeding time upon tail-tip transection is shown. All data represent the mean ± standard deviation. Statistical significance: *p < 0.05, **p < 0.01, ***p < 0.001 and *ns*,* p >* 0.05 (not significant).

Subendothelial collagen exposure causes sustained flow-resistant platelet adhesion, aggregation, and thrombus development after endothelial damage^1,2^. Under shear, NOX2-knockout mouse platelets exhibited decreased adherence and thrombus volume on collagen^52^. With DiOC6-stained platelets perfused in a collagen-coated flow chamber, we examined platelet adherence and thrombus development under shear. Platelets treated with GSK2795039 had less stable platelet adhesion and smaller platelet thrombi on immobilized collagen under shear (Fig. 8A).

We next tested the antithrombotic efficacy of GSK2795039 in vivo using two mouse models of thrombosis. Topical FeCl_3_ application to the carotid artery is a proven animal model for thrombosis and arterial injury^53^. Injuring the vascular endothelium with FeCl_3_ exposes collagen to platelet adhesion and thrombus, leading to endothelial denudation from ferric ion transendothelial migration^54^. Figure 8B shows that GSK2795039-administered animals protected against carotid occlusion better than vehicle controls, suggesting that GSK2795039 may delay thrombus formation in vivo. To further examine platelet-specific thrombotic potential, we utilized a pulmonary thromboembolism model induced by intravenous injection of collagen and epinephrine. Mice pretreated with GSK2795039 showed a dose-dependent increase in survival time compared to controls (Fig. 8C), supporting its protective role against platelet-driven thromboembolism.

Importantly, to assess whether GSK2795039 interferes with physiological hemostasis, we performed a tail bleeding time assay. Mice treated with GSK2795039 at either dose did not show a significant difference in bleeding time relative to vehicle-treated animals (Fig. 8D), indicating that the compound does not impair primary hemostasis at therapeutically effective doses.

These findings collectively demonstrate that GSK2795039 suppresses pathological thrombus formation under flow and in vivo, without compromising hemostatic function.

## Discussion

Our data, along with previous studies^14^, indicate that GSK2795039 does not affect thrombin- or U46619-induced human platelet aggregation but can inhibit collagen-induced platelet aggregation. This discrepancy may be due to the essential role of NOX2-derived ROS in collagen-mediated platelet activation and aggregation, whereas its impact on thrombin- or U46619-mediated responses is limited^7^. Studies demonstrating that GPVI stimulation significantly increases ROS production in human platelets, compared to much lower ROS levels induced by thrombin or U46619^15–17^, further highlight the critical role of NOX2-mediated ROS in platelet activation. However, in contrast, the studies by Vara et al.^6,55^ reported no significant inhibition of collagen-induced aggregation in NOX2^−/−^ platelets. These findings suggest that the properties of NOX may differ between human and mouse platelets, and further research is needed to clarify these differences.

Our findings demonstrate that GSK2795039 effectively suppresses platelet activation by attenuating NOX2-derived ROS generation and maintaining PTP activity. Although NOX2 was not genetically or immunologically isolated in this study, we employed two complementary biochemical approaches to assess NADPH oxidase activity in human platelets. The first assay measured extracellular superoxide production using L-012 chemiluminescence, while the second directly quantified NADPH oxidase enzymatic activity in platelet lysates. Both assays showed significant inhibition in response to GSK2795039, supporting its functional suppression of NADPH oxidase activity in human platelets (Fig. 1D and F). Given that GSK2795039 selectively inhibits NOX2 via competitive binding to its NADPH-binding site^13^, and that no effect was observed on aggregation induced by non-GPVI agonists (thrombin, U46619), the observed inhibition is most likely attributed to NOX2. Given that PTPs serve as key regulators of phosphorylation-dependent signaling and are vulnerable to redox-dependent inactivation^20,56^, preserving their function is essential for modulating platelet responses. Treatment with GSK2795039 led to a marked reduction in intracellular ROS levels in response to collagen (Fig. 1), and notably restored PTP enzymatic activity under oxidative conditions (Fig. 2B). As a result, the phosphorylation status of critical signaling mediators, including Syk, LAT, Vav1, Btk, and PLCγ2, was diminished, indicating impaired GPVI-mediated signal transduction (Figs. 2C and 3A). These data collectively suggest that GSK2795039 mitigates aberrant platelet activation by sustaining PTP function through redox regulation.

GPVI-PLCγ2 signaling activates IP_3_ receptor (IP_3_R) to release Ca^2+^ from the dense tubular system, like the endoplasmic reticulum in platelets. The sarcoendoplasmic reticulum and plasma membrane Ca^2+^-ATPases (SERCA and PMCA) recover cytosolic Ca^2+^ to near-resting levels after stimulation^57^. Collagen-stimulated platelets can produce H_2_O_2_ at 1 mM^15^. H_2_O_2_-induced cytosolic Ca^2+^ elevations in platelets are linked to sulfhydryl oxidation-dependent IP_3_R activation, SERCA inhibition, and PMCA inhibition^58^. Our findings suggest a model in which GSK2795039 inhibits collagen-induced elevation of extracellular H_2_O_2_ (Fig. 1), which can freely diffuse into the cytosol and increase cytosolic Ca^2+^ by oxidizing IP_3_R and SERCA and inhibiting PMCA’s ability to extrude Ca^2+^. Further research is needed to identify which of the various ROS generated by NOX2 in activated platelets regulates the modulator of intracellular Ca^2+^ levels in a redox-dependent manner.

Both plasma fibrinogen and fibrinogen released from α-granules bind to the activated integrin-αIIbβ3 on the platelet membrane, which is essential for platelet aggregate formation^47^. P-selectin, an α-granule membrane protein, moves to platelet membrane and interacts with P-selectin glycoprotein ligand-1 on the surface of the leukocytes or endothelial cells to generate thrombus in vivo^38^. Dense-granule factors, like ADP and epinephrine, stimulate platelets autocrinely and paracrinely. Consistent with its inhibitory effect on the GPVI-PLCγ2 signaling pathway, GSK2795039 also inhibits collagen-stimulated granule release. These findings suggest that the inhibitory effect of GSK2795039 on platelet-mediated thrombosis in vivo is largely attributed to its suppression of granule release, in addition to its inhibition of the GPVI-PLCγ2 signaling pathway.

Another mechanism contributing to the antiplatelet activity of GSK2795039 is its modulation of the NO/cGMP/PKG/VASP signaling axis. This pathway plays a crucial inhibitory role in platelet activation by suppressing integrin αIIbβ3 activation and aggregation through PKG-mediated phosphorylation of VASP at Ser239^49,59^. However, NOX2-derived ROS can scavenge NO to form peroxynitrite, thereby reducing NO bioavailability and impairing sGC activation and downstream cGMP signaling^60,61^. Our results show that GSK2795039 restores VASP phosphorylation and intracellular cGMP levels in collagen-stimulated platelets, indicating preservation of NO/sGC/PKG signaling by suppressing NOX2-derived ROS. These effects were PKG-dependent, unaffected by PKA inhibition, and mimicked by antioxidants, highlighting a redox-sensitive regulatory mechanism. Notably, reduced integrin αIIbβ3 activation and aggregation were observed in parallel, consistent with enhanced VASP phosphorylation. Thus, GSK2795039 appears to inhibit platelet aggregation not only by disrupting GPVI signaling but also by preserving the endogenous NO/cGMP/PKG inhibitory pathway through redox modulation. Lastly, we note that all experiments were conducted under continuous stirring to reflect physiological shear. This experimental design was chosen to recapitulate physiological shear environments necessary for ROS production by NOX2, as established by Xu et al.^52^. Consistent with previous reports showing that stirring alone can enhance VASP Ser^239^ phosphorylation in human platelets^62^, this condition likely accounts for the elevated baseline levels of VASP phosphorylation observed in our study.

Our findings indicate that the downregulation of the ROS-driven ASK1/p38 MAPK/cPLA_2_/TXA_2_ signaling pathway is associated with the antiplatelet action of GSK2795039. Additionally, GSK2795039 seems to inhibit TXA_2_ production independently of the ASK1/p38 axis. Activated by cytosolic Ca^2+^, cPLA_2_ translocates to membranes via a Ca^2+^-dependent phospholipid‐binding domain^63^. Thus, it is plausible that GSK2795039’s impact on TXA_2_ production may partially stem from a reduction in Ca^2+^-dependent cPLA_2_ activation, as it suppressed the rise in cytosolic Ca^2+^ in platelets stimulated with collagen. Notably, Sledz et al.^44^ showed that although ASK1 initiates p38 MAPK phosphorylation in human platelets, continued phosphorylation is driven by autophosphorylation through Syk. Given this, and GSK2795039’s inhibitory effect on Syk activation (Fig. 2), it is likely that GSK2795039 also contributes to the suppression of p38 MAPK/cPLA_2_/TXA_2_ signaling by inhibiting Syk-mediated autophosphorylation of p38 MAPK.

Moreover, we acknowledge that the inhibition of serine/threonine phosphorylation observed in Fig. 5 suggests broader redox-sensitive signaling modulation beyond tyrosine phosphatases. Collagen stimulation increases intracellular ROS, which activates ASK1 (MAP3K) via autophosphorylation at Thr^838^. Activated ASK1, in turn, phosphorylates and activates MKK3 (at Ser^189^) and MKK6 (at Ser^207^), both of which are dual-specificity MAPK kinases. These kinases subsequently phosphorylate p38 MAPK at Thr^180^/Tyr^182^, promoting its activation. Activated p38 MAPK then phosphorylates cPLA_2_ at Ser^510^, enhancing its enzymatic activity. The stepwise inhibition of these phosphorylation events by GSK2795039 strongly suggests suppression of the ROS–ASK1–MKK–MAPK signaling axis. Therefore, while PTP preservation may contribute to inhibition of tyrosine phosphorylation, GSK2795039 also appears to modulate serine/threonine kinase cascades downstream of NOX2-dependent ROS production.

The sustained and continuous adherence of platelets to the extracellular matrix is crucial for thrombus formation at sites of arterial injury^1,2^. Both intracellular and extracellular ROS are key factors that facilitate platelet adhesion and enhance platelet activation in response to shear stress, thereby promoting thrombus formation^6,7,52^. Given that GSK2795039 significantly reduces intracellular and extracellular ROS levels triggered by collagen (Fig. 1) and inhibits thrombus formation as well as platelet adhesion to collagen under shear conditions (Fig. 8A), it suggests that GSK2795039 may hinder stable platelet adhesion and thrombus progression within the vasculature of cardiovascular disease patients. This hypothesis is further supported by our in vivo results showing that GSK2795039 delays thrombus formation in a ferric chloride-induced carotid artery injury model and significantly improves survival in a pulmonary thromboembolism model (Fig. 8B and C). These data indicate that NOX2 inhibition by GSK2795039 confers protection against platelet-mediated arterial and pulmonary thrombosis. Notably, despite its antithrombotic efficacy, GSK2795039 did not prolong bleeding time in the tail transection assay (Fig. 8D), suggesting that it does not compromise primary hemostasis. This distinguishes GSK2795039 from conventional antiplatelet agents, which often increase bleeding risk by interfering with essential hemostatic mechanisms. The apparent dissociation between antithrombotic activity and hemostatic function may arise from the preferential targeting of NOX2-derived ROS, which are more critical in pathological thrombus propagation than in physiological platelet plug formation. These findings reinforce the potential of GSK2795039 as a novel therapeutic with a favorable safety profile.

However, the present study has several limitations. First, while GSK2795039 is a promising selective NOX2 inhibitor, its therapeutic development must focus on specificity and off-target effects. GSK2795039 lowers ROS production by competitively binding to NOX2’s NADPH binding site^13^. The selective inhibition of NOX2 over other NADPH oxidase isoforms is due to differences in the NADPH binding site, making GSK2795039 more specific. NOX inhibitors, such as GSK2795039, can have unintended side effects on other NADPH-dependent enzymes, making their development difficult. Because NADPH is required for antioxidant defence and metabolism, inhibitors may have an unintended effect on enzymes. Second, patients with X-linked chronic granulomatous disease are vulnerable to serious infections and immune disorders due to their genetic NOX2 deficiency. This implies that GSK2795039’s inhibition of NOX2 could potentially lead to unfavourable immunological adverse effects. Although the current study does not entirely eliminate this possibility, we do not expect that transient NOX2 inhibition will produce the same clinical outcomes as a permanent NOX2 absence, since the temporary and dose-dependent effects of GSK2795039 are likely to differ from those caused by a genetic NOX2 deficiency. Further studies are needed to evaluate the long-term safety of GSK2795039, particularly with respect to its impact on the immune system, to confirm its viability as a safe and effective antiplatelet therapy.

In conclusion, our work demonstrates that NOX2 inhibitor GSK2795039 is capable of inhibiting collagen-induced ROS generation and subsequent signaling events mediated by ROS, ultimately leading to attenuation of collagen-induced platelet aggregation and platelet-dependent thrombosis. These observations suggest that GSK2795039 may be potentially beneficial in the prevention of platelet-mediated thrombotic disorder.

## Materials and methods

### Ethics statements

All animal experiments were approved by the Institutional Animal Care and Use Committee of Seoul National University (Approval No. SNU-230731-2-3), and all methods were performed in accordance with relevant guidelines and regulations. The study is reported in accordance with the ARRIVE guidelines (https://arriveguidelines.org).

The collection and use of human blood samples from healthy volunteers were approved by the Seoul National University Institutional Review Board under Protocol No. 2206/001–006, and all methods involving human participants were conducted in accordance with the relevant guidelines and regulations. Informed consent was obtained from all participants.

### Reagents and antibodies

The study utilized several reagents, including GSK2795039, DCFH_2_, polyethylene glycol 300, Rp-8-pCPT-cGMPS, H-89, 8-pCPT-cGMP, (all from MedChem Express, Princeton, NJ, USA), CM-H_2_DCFDA, 3,3’-dihexyloxacarbocyanine Iodide (DiOC_6_), Fluo-3-AM (all from Molecular Probes, Eugene, OR, USA), 4-(2-aminoethyl)benzenesulfonyl fluoride (AEBSF) (Gold Biotechnology, St. Louis, MO, USA), aprotinin, NaCl, NaHCO_3_, Nonidet P-40, Tris (all from Amresco, Solon, OH, USA), CaCl_2_, catalase, (both from Calbiochem, San Diego, CA, USA), DMSO, EGTA, Evans blue, FeCl_3_, glucose, iodoacetamide, KH_2_PO_4_, Na_2_HPO_4_, Na_3_VO_4_, Na_4_P_2_O_7_•10H_2_O, NaF, N-ethylmaleimide, paraformaldehyde, prostaglandin E1, sodium citrate, thrombin, Triton X-100, β-glycerophosphate, Tween 80, U46619, NADPH, L-012, Tempol (all from Sigma-Aldrich, St. Louis, MO, USA), collagen (Chrono-Log, Havertown, PA, USA), citric acid (Duksan, Seoul, Korea), EDTA, KCl, leupeptin, MgCl_2_ (all from USB, Cleveland, OH, USA), dithiothreitol (Duchefa Biochemie, Haarlem, Netherlands), FITC-labelled annexin V (BD Biosciences, San Jose, CA, USA), HEPES (Thermo Fisher Scientific, Waltham, MA, USA), DEA-NONOate (Enzo Biochem, Farmingdale, NY, USA), Mn(III)TMPyP (Cayman, Ann Arbor, MI, USA).

All antibodies used in this study were used are summarized in Supplementary Table S1.

### Human platelet preparation

Blood samples from healthy, medication-free individuals were collected into tubes containing acid/citrate/dextrose (22.0 g sodium citrate, 24.5 g dextrose, and 7.3 g citric acid per 1 L) (Becton Dickson, Franklin, NJ, USA). Platelet-rich plasma (PRP) was separated by centrifugation using low speed centrifuge (Hanil Scientific Inc., Gimpo, Republic of Korea) at 150×*g* for 15 min, followed by an additional centrifugation at 300×*g* for 10 min at 25 °C using to concentrate the platelets. The pellet was resuspended in Tyrode’s-HEPES buffer (10 mM HEPES [pH 7.4], 129 mM NaCl, 0.8 mM KH_2_PO_4_, 8.9 mM NaHCO_3_, 2.8 mM KCl, 0.8 mM MgCl_2_, and 5.6 mM glucose) supplemented with 2 mM EDTA, 10% of citric acid/citrate/dextrose solution, and 1 µM prostaglandin E1, and washed once more. Washed platelets were resuspended in Tyrode’s-HEPES buffer at the appropriate concentration. Unless specified, washed platelets were treated with 1 mM CaCl_2_ for 2 min prior to stimulation.

### Light transmission aggregometry

Platelet aggregation was assessed by measuring changes in light transmission using a 4-channel aggregometer (Chrono-Log, Havertown, PA, USA) at 37 °C under continuous stirring (1000 rpm). Data were recorded using Aggrolink software, with 100% transmission calibrated using buffer and washed platelets for the respective controls.

### NOX activity assay

NOX activity was determined as previously described^64^. Briefly, washed platelet lysates (10 µg protein) were incubated with 100 µM L-012 and 0.5 mM NADPH at 37 °C in the dark, and luminescence was detected using a luminometer (Berthold Technologies, Oak Ridge, TN, USA). NOX activity was first calculated as relative luminescence units (RLU) per µg protein, and the results were presented as relative fold changes compared to the control group.

### Assessment of intracellular ROS and cytosolic Ca^2+^ levels

For the measurement of intracellular ROS and Ca^2+^ levels, washed platelets (5 × 10^8^/mL) were loaded with CM-H_2_DCFDA (5 µM) or Fluo-3-AM (1 µM) by incubation at 37 °C for 30 min in the dark. At 37 °C, a fluorometer cuvette was used to stimulate platelets (5 × 10^8^/mL) in Tyrode’s-HEPES buffer with constant stirring at 1000 rpm. A spectrofluorophotometer (Jasco, Tokyo, Japan) measured the fluorescence of CM-DCF (495 nm excitation, 525 nm emission) or Fluo-3 (488 nm excitation, 525 nm emission).

### Assessment of extracellular ROS levels

Washed platelets (5 × 10^8^/mL) in Tyrode’s-HEPES buffer with 1 µM DCFH_2_ were stimulated with 10 µg/mL collagen in a fluorometer cuvette at 37 °C with continual stirring at 1000 rpm. The fluorescence of DCF (488 nm excitation, 525 nm emission) was measured using a spectrofluorophotometer as described above.

### Assessment of extracellular superoxide anion levels

Extracellular superoxide anion levels were assessed using L-012, a chemiluminescent probe specific for superoxide^65^. After washed platelets (5 × 10^8^/mL) in Tyrode’s-HEPES buffer containing 100 µM L-012 were stimulated, luminescence was read immediately 30-second intervals over 5 min using a luminometer (Berthold Technologies, Oak Ridge, TN, USA).

### Assessment of extracellular H_2_O_2_ levels

The Amplex Red hydrogen peroxide/peroxidase assay kit (Invitrogen, New York, NY, USA) was used. Washed platelets (5 × 10^8^/mL) were treated at 1000 rpm at 37 °C in a Thermomixer (Eppendorf, Hamburg, Germany). The supernatant (50 µL) obtained by centrifugation using micro centrifuge (Hanil Scientific Inc., Gimpo, Republic of Korea) at 12,000×*g* for 30 s at 25 °C was added to each black 96-well containing a reaction mixture (50 µL) containing horseradish peroxidase (0.2 U/mL) and Amplex Red reagent (100 µM) to start the reaction. After 30 min of incubation, a Cytation3 microplate reader (BioTek, Burlington, VT, USA) detected fluorescence (520 nm excitation, 590 nm emission).

### PTP activity assay

PTP activity was measured as described previously^66^. Washed platelets were stimulated under experimental conditions and lysed in an oxygen-deprived environment (< 1% O₂; Mbraun glovebox workstation, Garching, Germany) using a buffer (50 mM Tris-HCl, pH 6.5, 150 mM NaCl, 1% Nonidet P-40) supplemented with 1 µg/mL leupeptin, 1 µg/mL aprotinin, 1 mM AEBSF, 10 mM iodoacetamide, and 10 mM N-ethylmaleimide to preserve the reduced state of catalytic cysteines. Samples were centrifuged using micro centrifuge (Hanil Scientific Inc., Gimpo, Republic of Korea) at 12,500×*g* for 10 min at 4 °C to remove cellular debris. The supernatant was incubated at room temperature for 30 min in the dark to allow complete alkylation of free thiol groups. Residual alkylating agents were quenched by the addition of 50 mM dithiothreitol. Protein concentration was then determined using the Bradford assay (Bio-Rad, Hercules, CA, USA). PTP enzymatic activity was measured using a commercially available colorimetric PTP assay kit (EMD Millipore, Billerica, MA, USA) according to the manufacturer’s instructions. Liberated inorganic phosphate was quantified by absorbance measurement, and PTP activity was normalized and expressed as nanomoles of phosphate released per minute per milligram of total protein.

### Western blotting

After stimulation, washed platelets were lysed in cell extraction buffer (20 mM HEPES [pH 7.0], 150 mM NaCl, 1% Triton X-100, 10% glycerol, 1 mM EDTA, 2 mM EGTA, 20 mM β-glycerophosphate, 1 mM Na_3_VO_4_, 1 µg/mL leupeptin, 1 µg/mL aprotinin, and 1 mM AEBSF). Lysates were centrifuged using micro centrifuge (Hanil Scientific Inc., Gimpo, Republic of Korea) at 12,000×*g* for 10 min at 4 °C to remove debris, and total protein concentrations were determined using the Bio-Rad protein assay dye reagent (Bio-Rad Laboratories, Richmond, CA). Equal amounts of protein (20–40 µg) were loaded per lane and subjected to SDS-PAGE. Western blot analysis was performed using specific antibodies, and band intensity was quantified with ImageJ software (NIH, Bethesda, MD, USA).

### TXB_2_ ELISA

Washed platelets (5 × 10⁸/mL) were stimulated and the reaction was halted by adding EDTA (5 mM) and indomethacin 200 µM). The supernatant was collected, and TXB₂ levels were measured using an ELISA kit (Cayman, Ann Arbor, MI, USA) and a Cytation3 microplate reader.

### Flow cytometry and analysis

Flow cytometry was performed to assess P-selectin exposure, integrin activation, and PS exposure using CD62P-PE (0.5 µg/mL) and PAC1-FITC (5 µg/mL) antibodies and annexin V-FITC (0.1 mg/mL), respectively. Washed platelets were fixed in 1% paraformaldehyde, diluted with PBS, and analyzed using a FACSCalibur flow cytometer, with data processed in FlowJo software (FlowJo LLC, Ashland, OR, USA).

### ATP release assay

Collagen-stimulated washed platelets were analyzed for ATP release using a ATP bioluminescent assay kit (Sigma-Aldrich, St. Louis, MO, USA) in a white 96-well plate. Luminescence was monitored at 30-second intervals over 5 min using an LB 960 Centro microplate luminometer (Berthold, Bad Wildbad, Germany).

### cGMP ELISA

Following centrifugation using micro centrifuge (Hanil Scientific Inc., Gimpo, Republic of Korea) at 12,000×*g* for 30 s at 4 °C, washed platelets were lysed in 0.1 M HCl with 1% Triton X-100. After additional centrifugation using micro centrifuge (Hanil Scientific Inc., Gimpo, Republic of Korea) at 12,000×*g* for 10 min at 4 °C, cGMP levels in the supernatant were quantified using an ELISA kit (Enzo Life Sciences, Exeter, UK) and a Cytation3 microplate reader.

### Determination of platelet adhesion and thrombus formation under flow conditions

As previously described^64^, DiOC_6_-labeled washed platelets were perfused over a collagen-coated surface in a flow chamber (Chamlide CF) at 1000 s⁻¹. Adherent platelets were fixed with paraformaldehyde and visualized under a fluorescence microscope. Image analysis for surface coverage was performed with ImageJ software.

### Animal Preparation and drug administration

C57BL/6J male mice (10–11 weeks old) were obtained from the Jackson Laboratory (Bar Harbor, ME, USA) and maintained in a specific-pathogen-free animal facility at Seoul National University. Mice were housed under controlled environmental conditions and fed a standard chow diet (Harlan Teklad, Madison, WI, USA) *ad libitum.* Mice were randomly divided into three experimental groups. GSK2795039 was orally administered at a dose of 10 mL/kg body weight via oral gavage in a vehicle composed of 20% DMSO, 20% Tween 80, and 60% polyethylene glycol 300, as previously described^13^. Control animals received an equivalent volume of the vehicle alone. One hour after GSK2795039 administration, mice were anesthetized by intraperitoneal injection of alfaxalone (100 mg/kg; Jurox, New South Wales, Australia) and xylazine (10 mg/kg; Rompun, Bayer Korea, Seoul, Korea).

### Pulmonary thromboembolism model

To induce pulmonary thromboembolism, a mixture of collagen (0.8 mg/kg; Type I, bovine, Sigma-Aldrich) and epinephrine (60 µg/kg; Sigma-Aldrich) dissolved in sterile saline was retro-orbitally injected in a final volume of 100 µL using a 30G insulin syringe. After injection, mice were returned to a warmed cage and continuously monitored for respiratory distress, paralysis, or death for up to 15 min. Mice exhibiting severe respiratory symptoms or sudden death were recorded as thromboembolism-positive. Time to death by pulmonary embolism was analyzed and utilized as a measure of susceptibility to thrombosis.

### Carotid occlusion model

To assess arterial thrombosis the right common carotid artery was surgically exposed. A Perivascular Flowprobe (model MA0.5PSB, Transonic Systems Inc., Ithaca, NY, USA) connected to a Perivascular Flowmeter (model TS420) was positioned around the isolated artery and calibrated to baseline flow (0.3–0.9 mL/s). Vascular injury was induced by applying a filter paper (1 × 1 mm) soaked in 20% FeCl₃ to the surface of the artery for 3 min. The filter paper was then removed, and blood flow was continuously recorded every 0.5 s. Stable occlusion was defined as complete cessation of flow (0 mL/second) sustained for at least 30 s.

### Tail bleeding assay

Hemostatic function was assessed using a tail bleeding time assay. The tail was transected at a fixed diameter of 1.5 mm from the tip using a sterile scalpel, and the remaining portion was immediately immersed in prewarmed isotonic saline at 37 °C. Bleeding was monitored visually, and the time to complete cessation of bleeding was recorded. Cessation was defined as no rebleeding for at least 1 min following the initial stop.

### Statistical analysis

Data were statistically analyzed using GraphPad PRISM software 10 (GraphPad Software Inc., La Jolla, CA, USA). The Shapiro–Wilk test was used for normality testing. Statistical significance was calculated using the t-test for two-group comparisons and one-way or two-way ANOVA tests for multiple comparisons with Bonferroni correction, respectively. *P* values less than 0.05 were considered statistically significant.

## Supplementary Information

Below is the link to the electronic supplementary material.

Supplementary Material 1

## Data Availability

The datasets generated during and/or analysed during the current study are available from the corresponding author on reasonable request.
