# Supplementary material for: NADPH oxidase 2 inhibitor GSK2795039 exerts antiplatelet and antithrombotic activity
Source: Sci Rep. 2025 Oct 16;15:36270. doi: 10.1038/s41598-025-20250-z (PMC12533013; doi:10.1038/s41598-025-20250-z)

Supplemental material

NADPH oxidase 2 inhibitor GSK2795039 exerts antiplatelet and antithrombotic activity

Eun Bee Oh[a,#], Yun Jeong Kong[b,#], Taeil Kim[a,#], Joara Jang[a], Hyunseong Yu[a], Ji Won Park[a], Solee Kim[a], Taeryeong Kim[a], Jisue Sohn[a] and Tong-Shin Chang[a,c,*]

[a]*College of Pharmacy, Seoul National University, Seoul 08826, Republic of Korea; ombi1202@snu.ac.kr, kti0594@snu.ac.kr, ara4030@snu.ac.kr, gustjd987@snu.ac.kr, jw3611@snu.ac.kr, soleezzang@snu.ac.kr, taeryeong@snu.ac.kr;*

[b]*Graduate School of Pharmaceutical Sciences, Ewha Womans University, Seoul 03760, Republic of Korea; kongd246@gmail.com*

[c]*Research Institute of Pharmaceutical Sciences, Seoul National University, Seoul 08826, Republic of Korea;*

* Correspondence: changts@snu.ac.kr; Tel.: +82-2-880-7852

# These authors contributed equally to this work.

Contents:

Supplementary Table S1 and legends

Supplemental Figures S1-S4 and Figure legends

Supplementary Table S1. Antibodies used in this study.

Antibody	Host	Company	Catalog #	Dilution
Btk	Rabbit	Santa Cruz Biotechnology	sc-1696	1:1000
cPLA$_2$	Rabbit	Cell Signaling Technology	2832	1:1000
CD62P-phycoerythrin	Mouse	BD Biosciences	555524	1:100
ERK5	Rabbit	Cell Signaling Technology	3552s	1:1000
LAT	Mouse	Santa Cruz Biotechnology	sc-53550	1:1000
MKK6	Rabbit	Cell Signaling Technology	9264S	1:1000
mouse IgG-horseradish peroxidase	Goat	Seracare Life Sciences	5220-0341	1:5000
p38 MAPK	Rabbit	Cell Signaling Technology	9212S	1:1000
p47phox	Mouse	Santa Cruz Biotechnology	sc-17844	1:1000
PAC1-fluorescein	Mouse	BD Biosciences	340507	1:100
phospho-ASK1 (Thr845)	Rabbit	Cell Signaling Technology	3765S	1:1000
phospho-Btk (Tyr551)	Rabbit	Invitrogen	44-1355G	1:1000
phospho-cPLA$_2$ (Ser505)	Rabbit	Cell Signaling Technology	2831	1:1000
phospho-ERK5 (Thr218/Tyr220)	Rabbit	Cell Signaling Technology	3371s	1:1000
phospho-LAT (Tyr200)	Rabbit	Abcam	ab68139	1:1000
phospho-MKK3 (Ser189)/MKK6 (Ser207)	Rabbit	Cell Signaling Technology	9236S	1:1000
phospho-p38 MAPK (Thr180/Tyr182)	Rabbit	Cell Signaling Technology	9236s	1:1000
phospho-p47phox (Ser304)	Rabbit	Invitrogen	PA5105496	1:1000
phospho-PLCγ2 (Tyr753)	Rabbit	Santa Cruz Biotechnology	sc-101785	1:1000
phospho-Syk (Tyr525/526)	Rabbit	Cell Signaling Technology	2711s	1:1000
phospho-VASP (Ser239)	Rabbit	Cell Signaling Technology	3114s	1:1000
phospho-Vav1 (Tyr174)	Rabbit	Santa Cruz Biotechnology	sc-16408-R	1:1000
phosphotyrosine (4G10)	Mouse	Sigma-Aldrich	05-321	1:1000
PLCγ2	Rabbit	Santa Cruz Biotechnology	sc-407	1:1000
Syk	Mouse	Santa Cruz Biotechnology	sc-1240	1:1000
rabbit IgG-horseradish peroxidase	Goat	Seracare Life Sciences	5220-0458	1:5000
VASP	Mouse	Santa Cruz Biotechnology	sc-46668	1:1000
Vav1	Rabbit	Abcam	ab40875	1:1000
β-actin	Rabbit	Abfrontier	LF-PA0207	1:1000

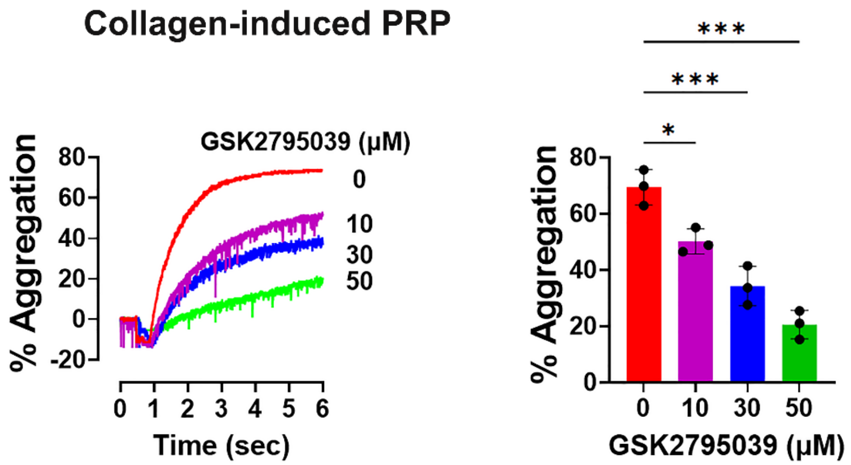

Supplementary Figure S1. GSK2795039 inhibits collagen-stimulated platelet aggregation in PRP. Platelet-rich plasma (PRP; 5 × 10^8/mL) was pretreated with vehicle (0.5% DMSO) or various concentrations of GSK2795039 for 5 minutes before stimulation with collagen (10 μg/mL). Platelet aggregation was monitored by measuring changes in light transmission, with 100% transmission defined as the buffer control. Data represent the mean ± standard deviation. Statistical significance: ** $p < 0.01$ and *** $p < 0.001$.

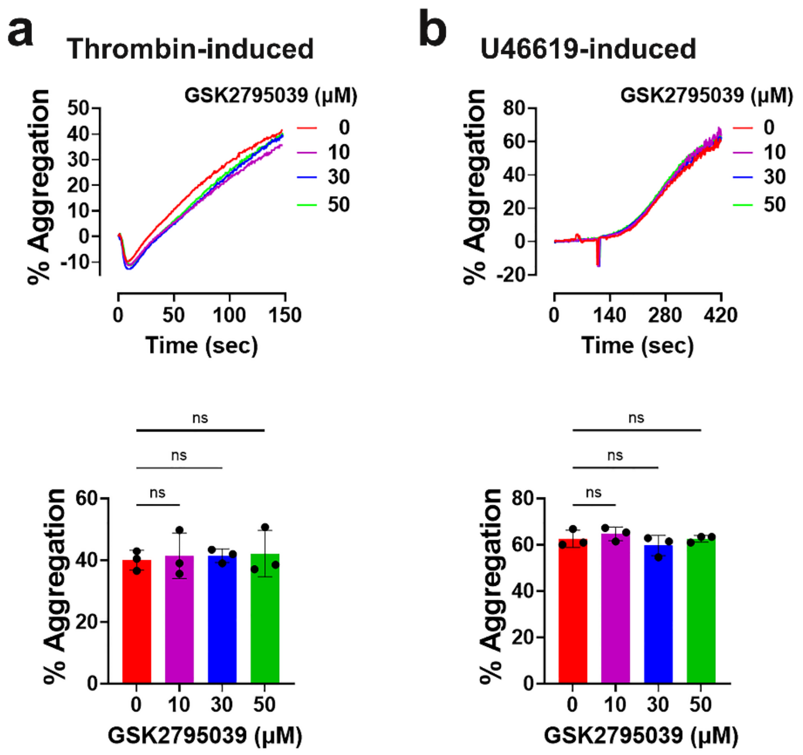

Supplementary Figure S2. GSK2795039 does not affect platelet aggregation induced by thrombin or U46619. Washed human platelets (5 × 10^8/mL) were treated with vehicle (0.5% DMSO) or GSK2795039 at the indicated concentrations for 5 minutes and stimulated with thrombin (0.1 U/mL; panel A) or U46619 (1 µM; panel B). Platelet aggregation was monitored by measuring changes in light transmission, with 100% transmission defined as the buffer control. Data represent the mean ± standard deviation from three independent donors. Statistical analysis indicates no significant difference (***ns***, $p > 0.05$).

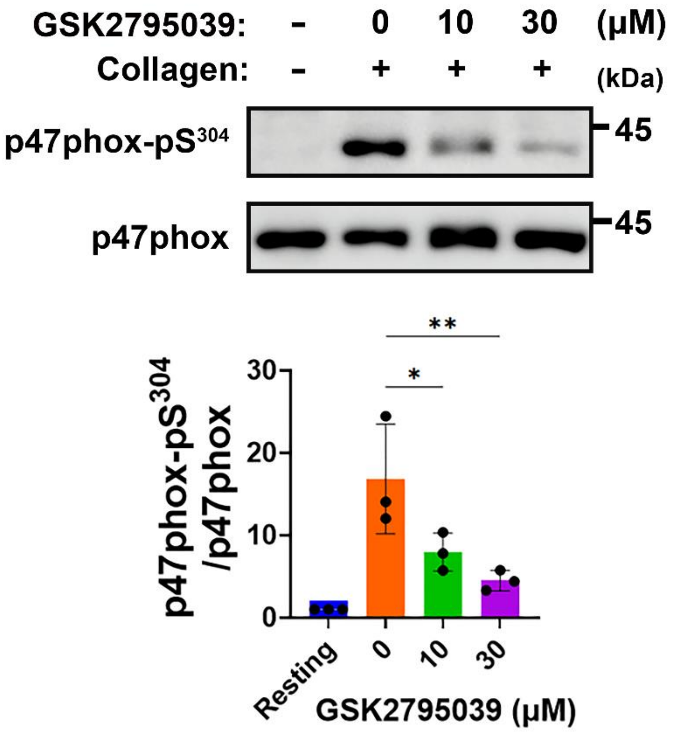

Supplementary Figure S3. GSK2795039 inhibits collagen-induced phosphorylation of p47phox. Immunoblot analysis for phosphorylation of p47phox at the Ser^{304} residue. The upper panels show representative immunoblot analysis with positions of molecular weight (kDa) markers as indicated. The quantitative data represent the mean ± standard deviation. Statistical significance: *$p < 0.05$ and **$p < 0.01$.

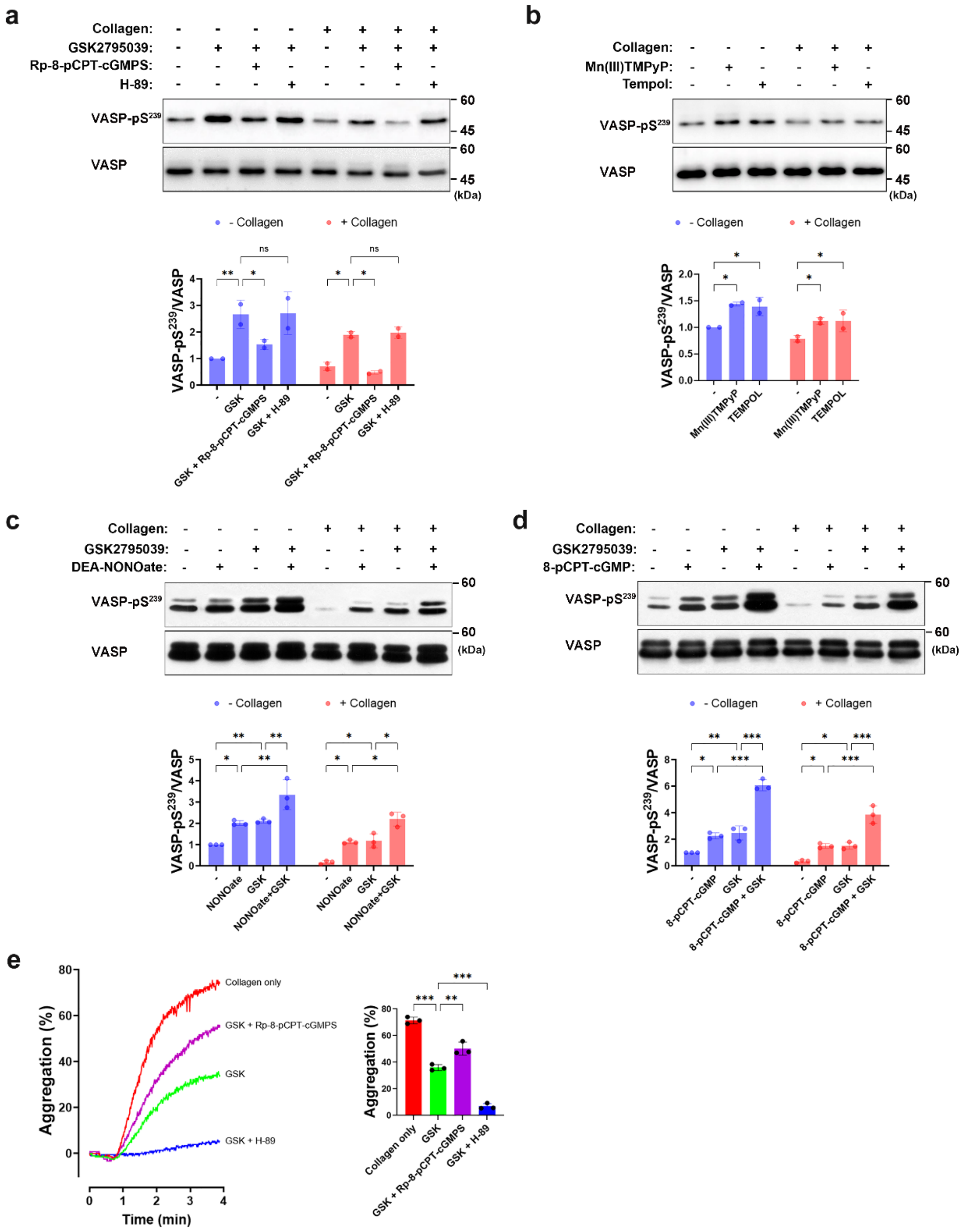

Supplementary Figure S4. GSK2795039 modulates the cGMP/PKG/VASP/aggregation signaling pathway in collagen-stimulated platelets. (A) Washed human platelets were preincubated with PKG inhibitor Rp-8-pCPT-cGMPS (50 µM) or PKA inhibitor H-89 (10 µM), in the presence or absence of GSK2795039 (30 µM) for 10 minutes, then stimulated with collagen. (B) Platelets were pretreated with antioxidants Mn(III)TMPyP or Tempol (1 mM each) for 10 minutes before collagen stimulation. (C) Platelets were treated with DEA-NONOate (50 µM) with or without GSK2795039 (30 µM) for 5 minutes followed by collagen stimulation for 2 minutes. (D) Platelets were pretreated with 8-pCPT-cGMP (50 µM) with or without GSK2795039 (30 µM) for 10 minutes prior to collagen stimulation. For panels A-D, VASP Ser^{239} phosphorylation was assessed by Western blot. Representative blots and densitometric analyses are shown. (E) Platelets were treated as in (A), and collagen-induced aggregation was measured by light transmission aggregometry. Platelet aggregation was monitored by measuring changes in light transmission, with 100% transmission defined as the buffer control. All data represent the mean ± standard deviation. Statistical significance: $*p < 0.05$, $**p < 0.01$, $***p < 0.001$ and *ns*, $p > 0.05$ (not significant).

Full uncropped immunoblots of Figure 2A.
The repeat 1 is used in Figure 2A.

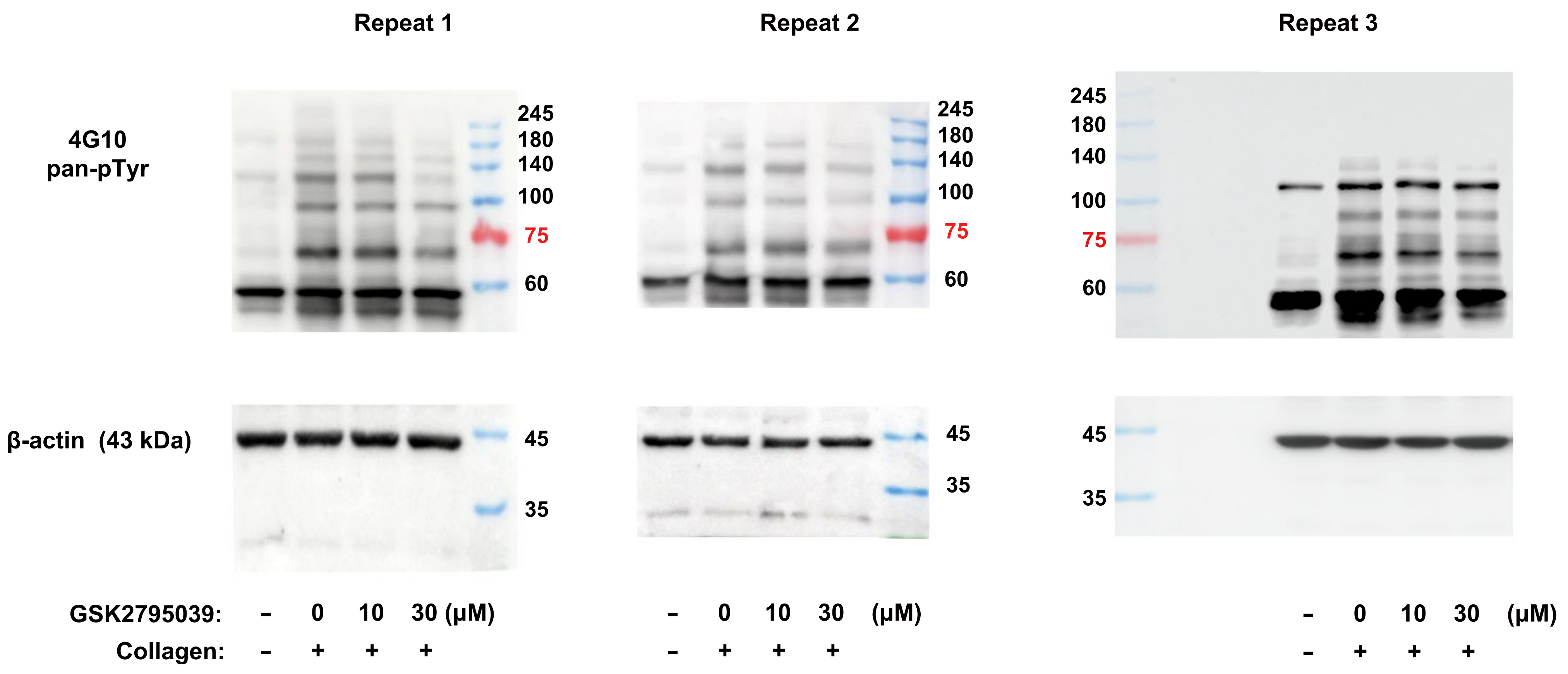

Full uncropped immunoblots of Figure 2C.
The repeat 1 is used in Figure 2C.

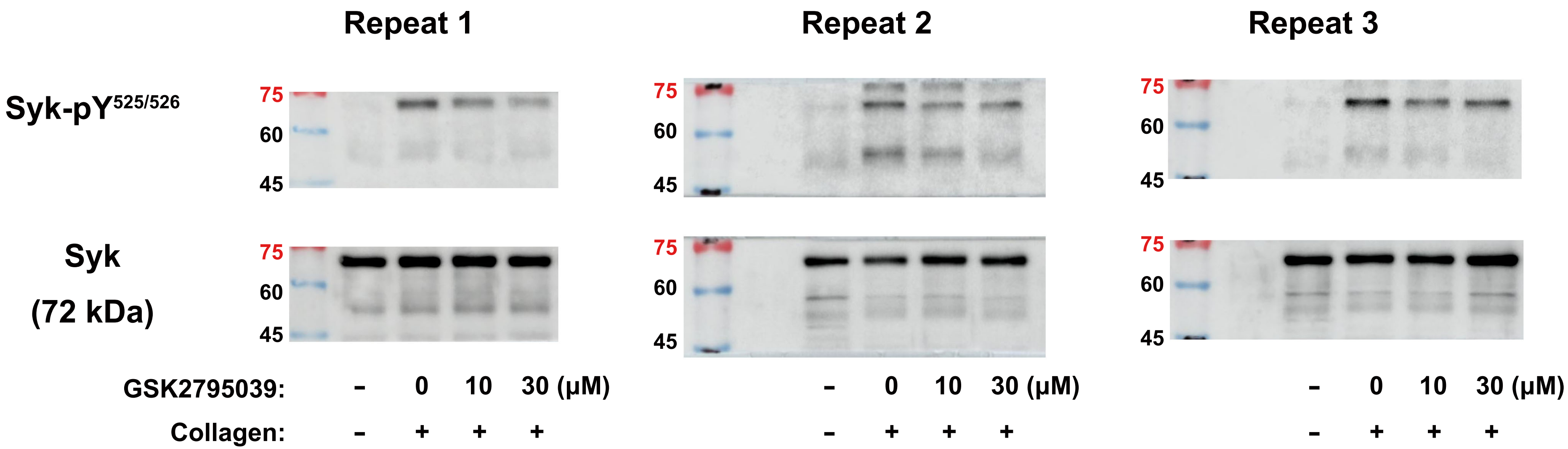

Full uncropped immunoblots of Figure 2C.
The repeat 1 is used in Figure 2C.

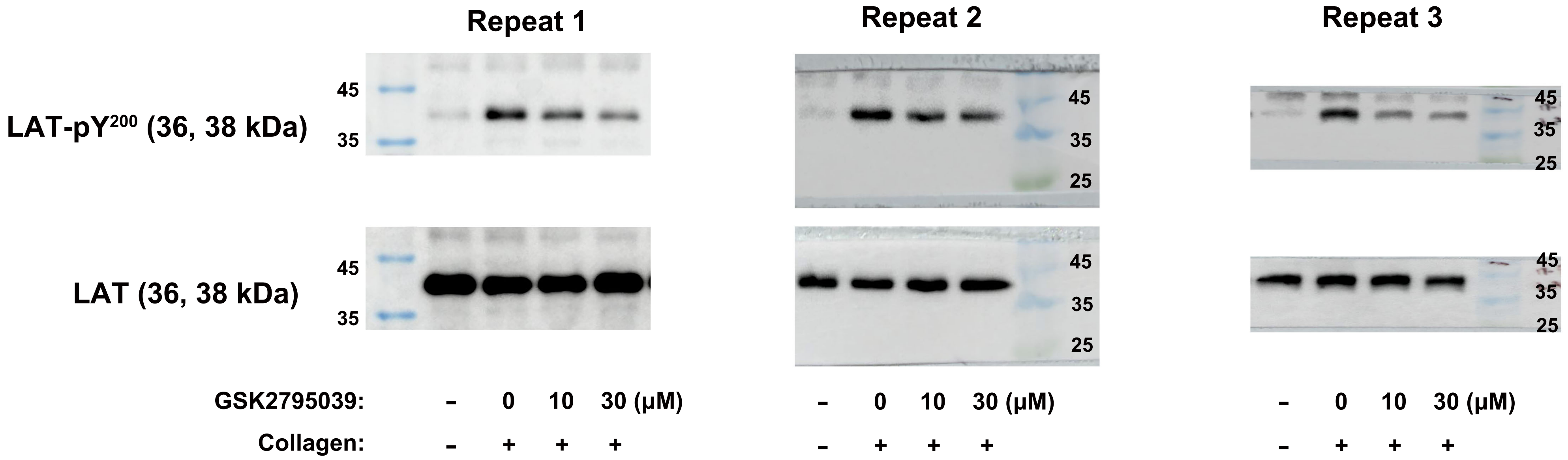

Full uncropped immunoblots of Figure 2C.
The repeat 1 is used in Figure 2C.

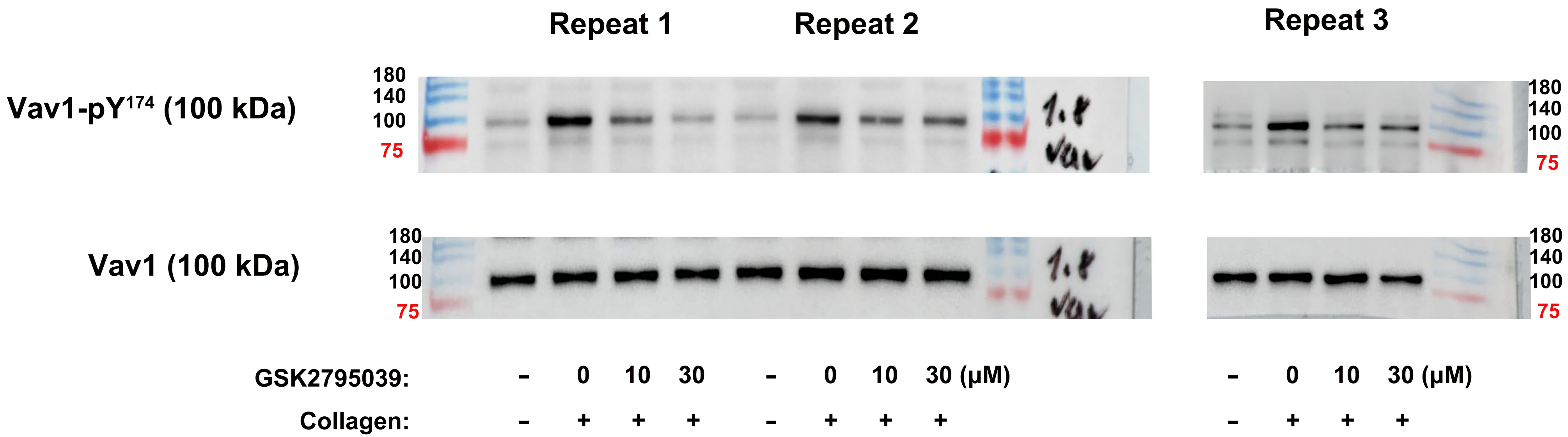

Full uncropped immunoblots of Figure 2C.
The repeat 1 is used in Figure 2C.

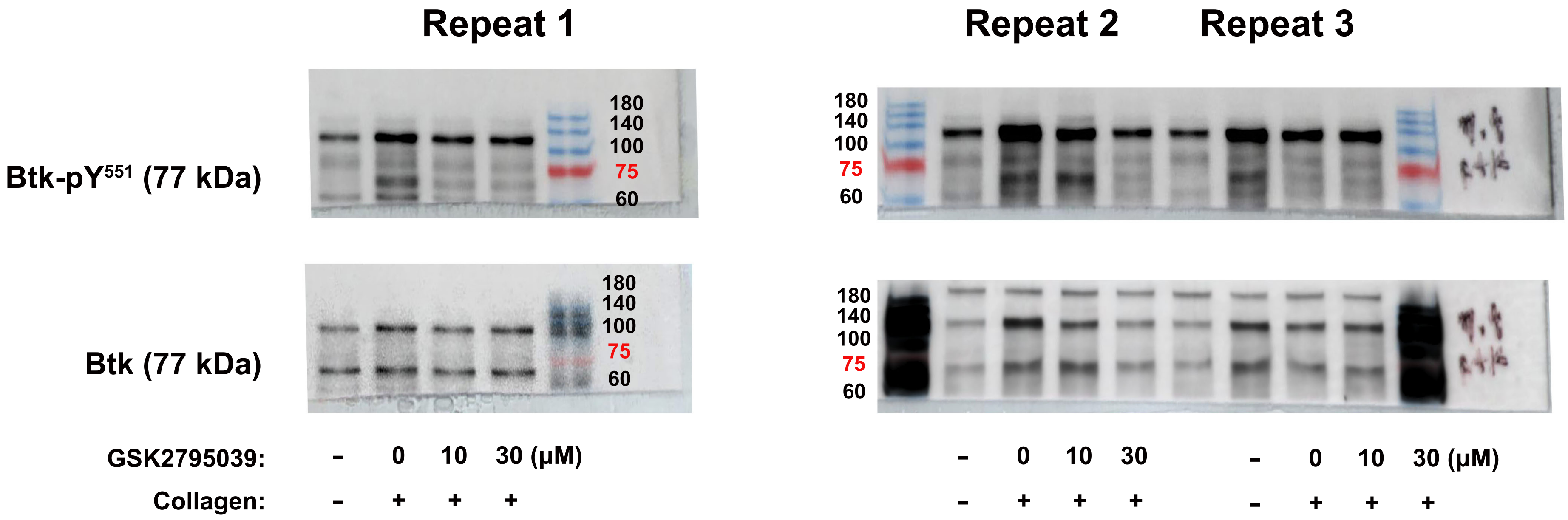

Full uncropped immunoblots of Figure 3A.
The repeat 1 is used in Figure 3A.

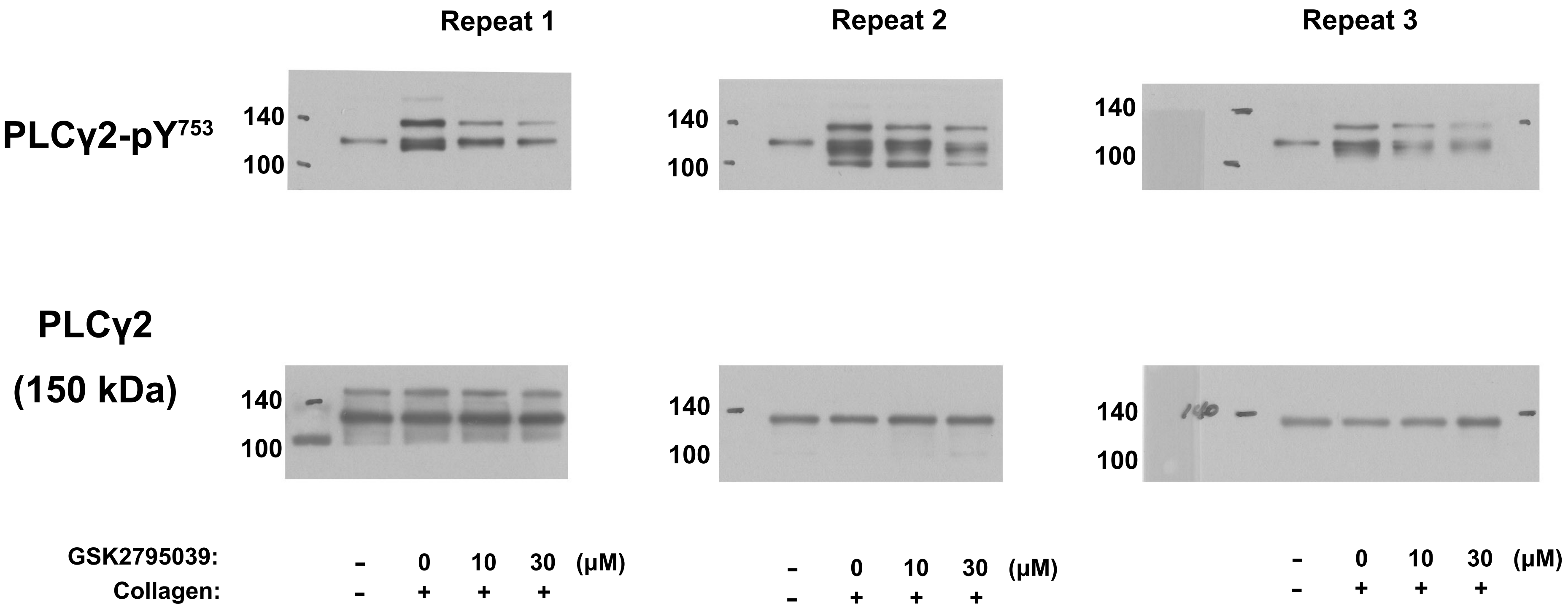

Full uncropped immunoblots of Figure 3B.
The repeat 1 is used in Figure 3B.

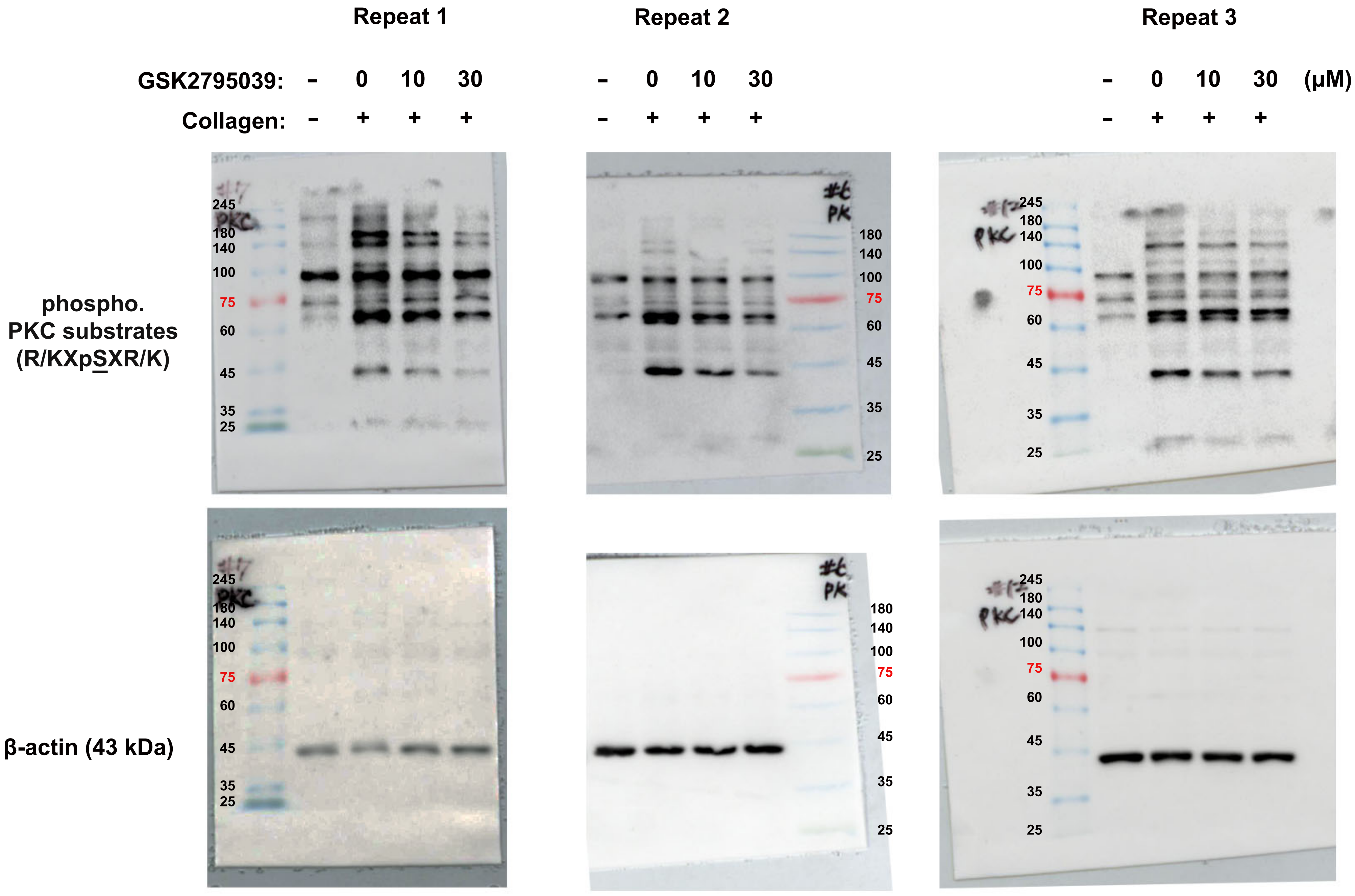

Full uncropped immunoblots of Figure 5A.
The repeat 1 is used in Figure 5A.

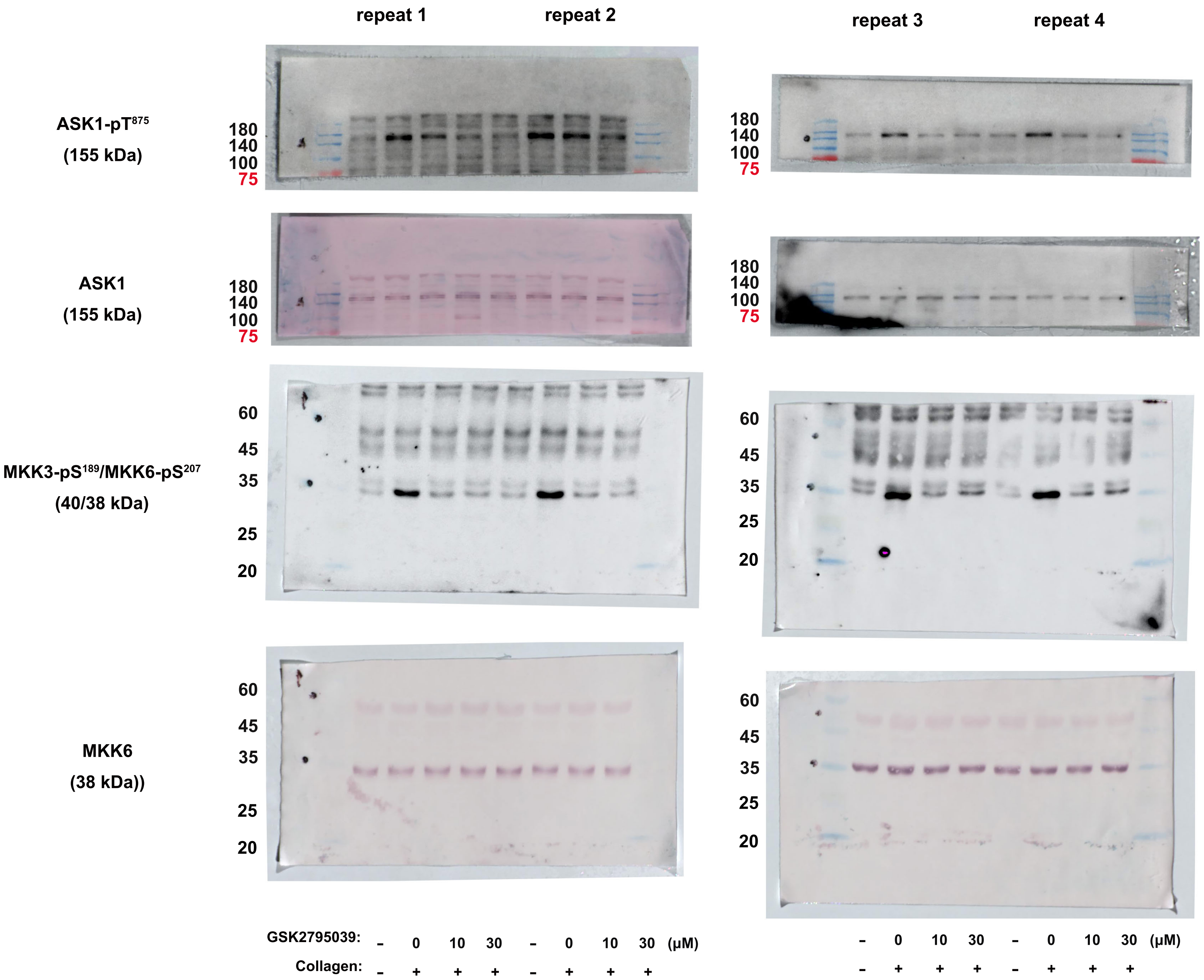

Full uncropped immunoblots of Figure 5A.
The repeat 1 is used in Figure 5A.

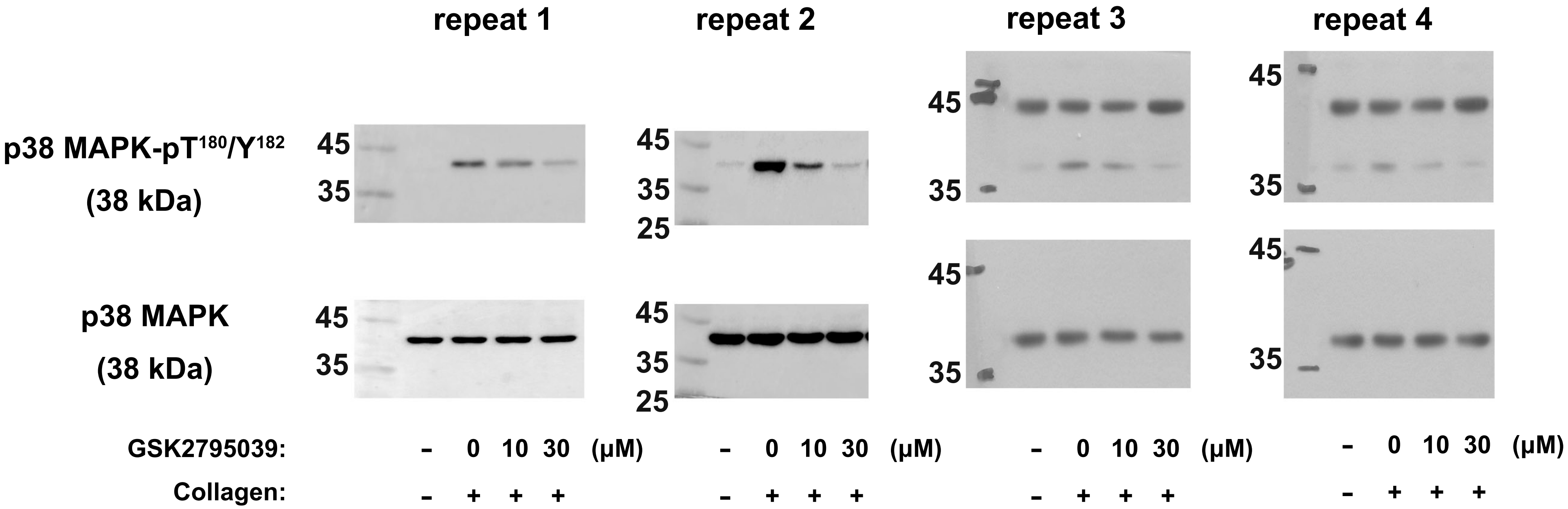

Full uncropped immunoblots of Figure 5B.
The repeat 3 is used in Figure 5B.

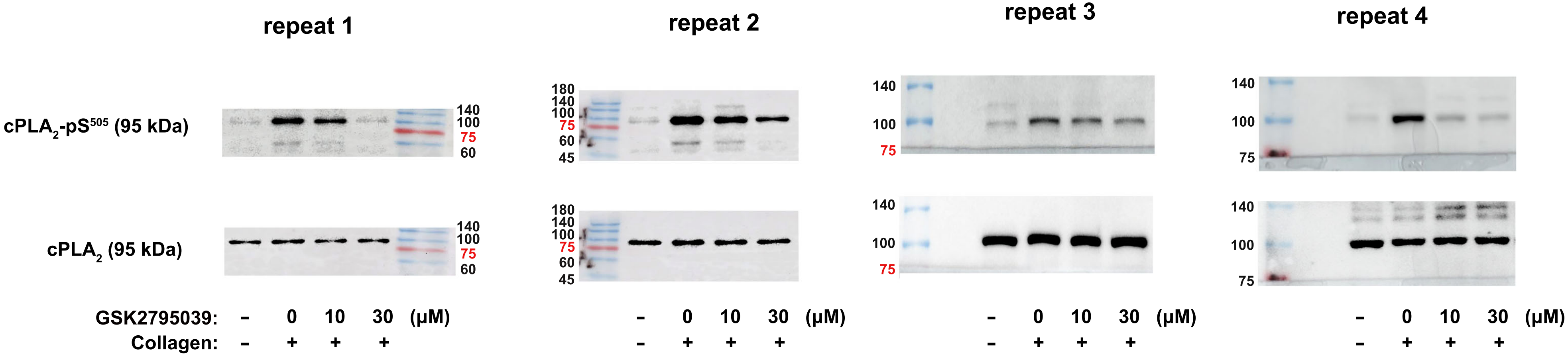

Full uncropped immunoblots of Figure 6A.
The repeat 1 is used in Figure 6A.

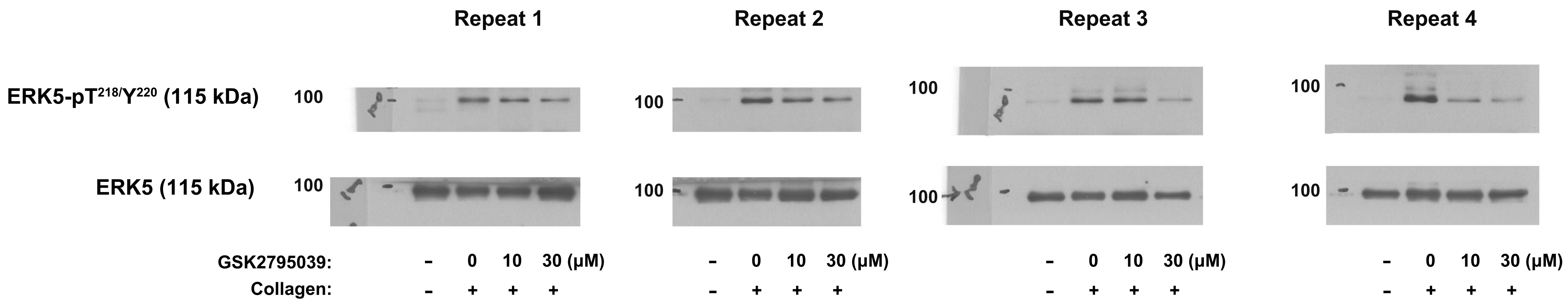

Full uncropped immunoblots of Figure 7B.
The repeat 1 is used in Figure 7B.

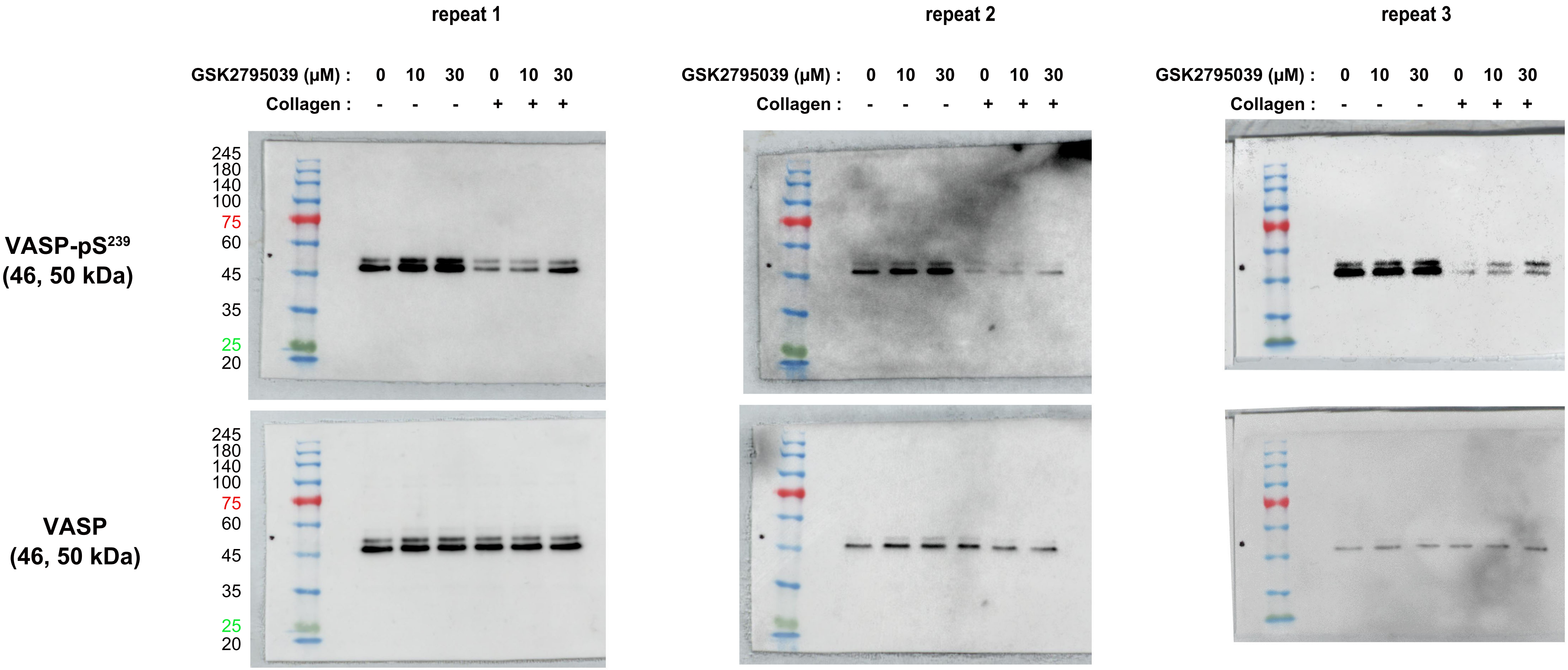

Full uncropped immunoblots of Supplementary Figure S3.
The repeat 1 is used in Supplementary Figure S3.

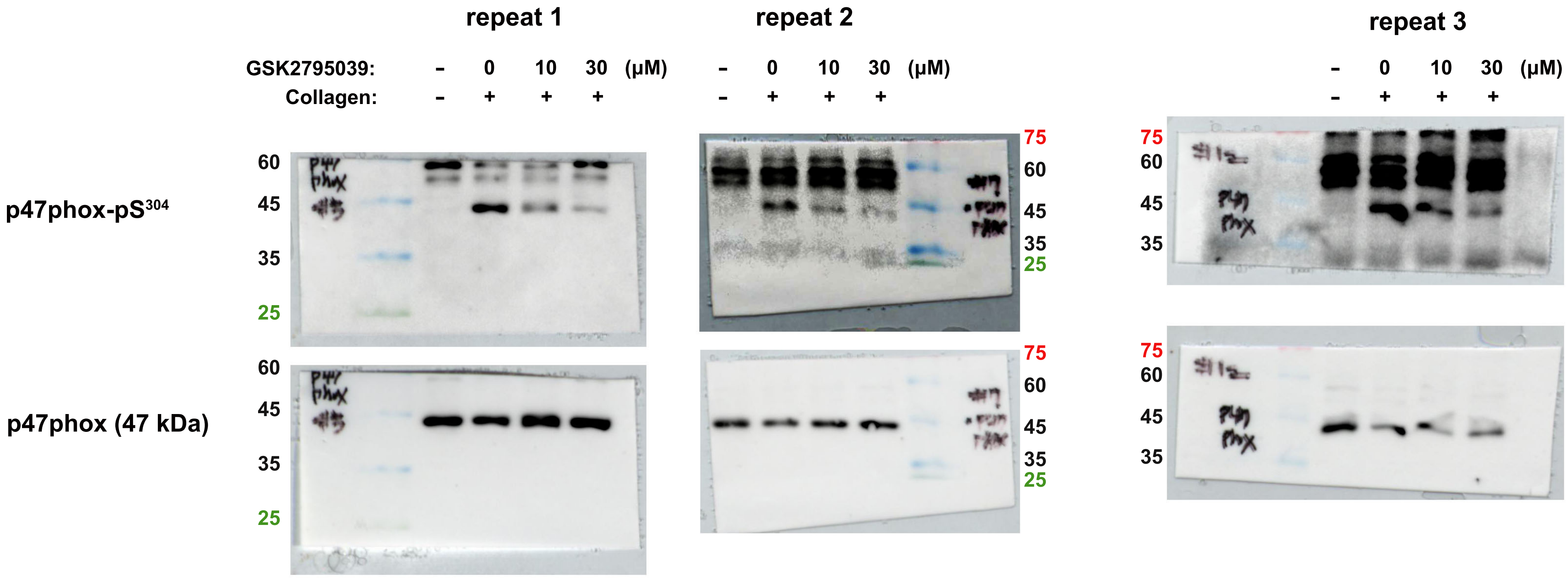

Full uncropped immunoblots of Supplementary Figure S4A.
The repeat 2 is used in Supplementary Figure S4A.

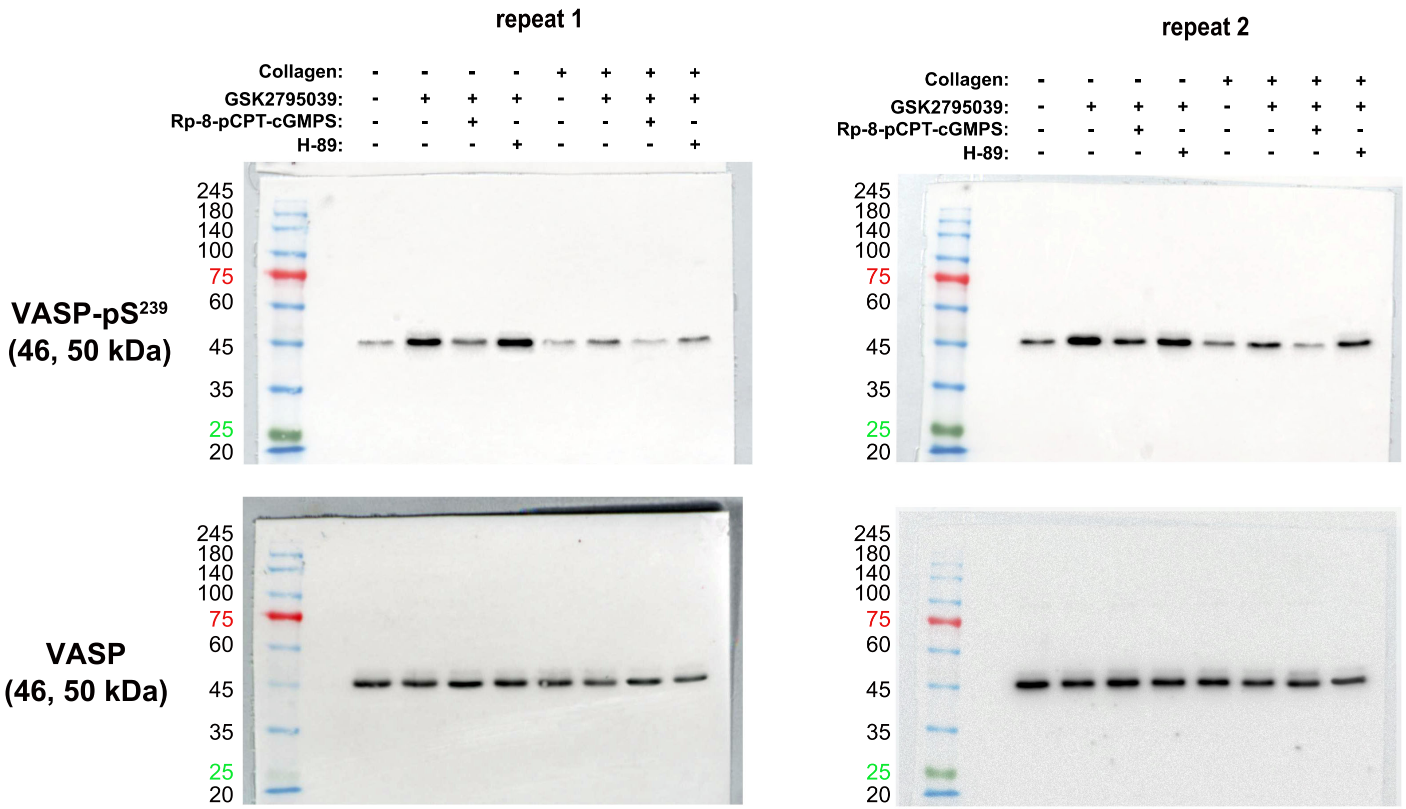

Full uncropped immunoblots of Supplementary Figure S4B.
The repeat 1 is used in Supplementary Figure S4B.

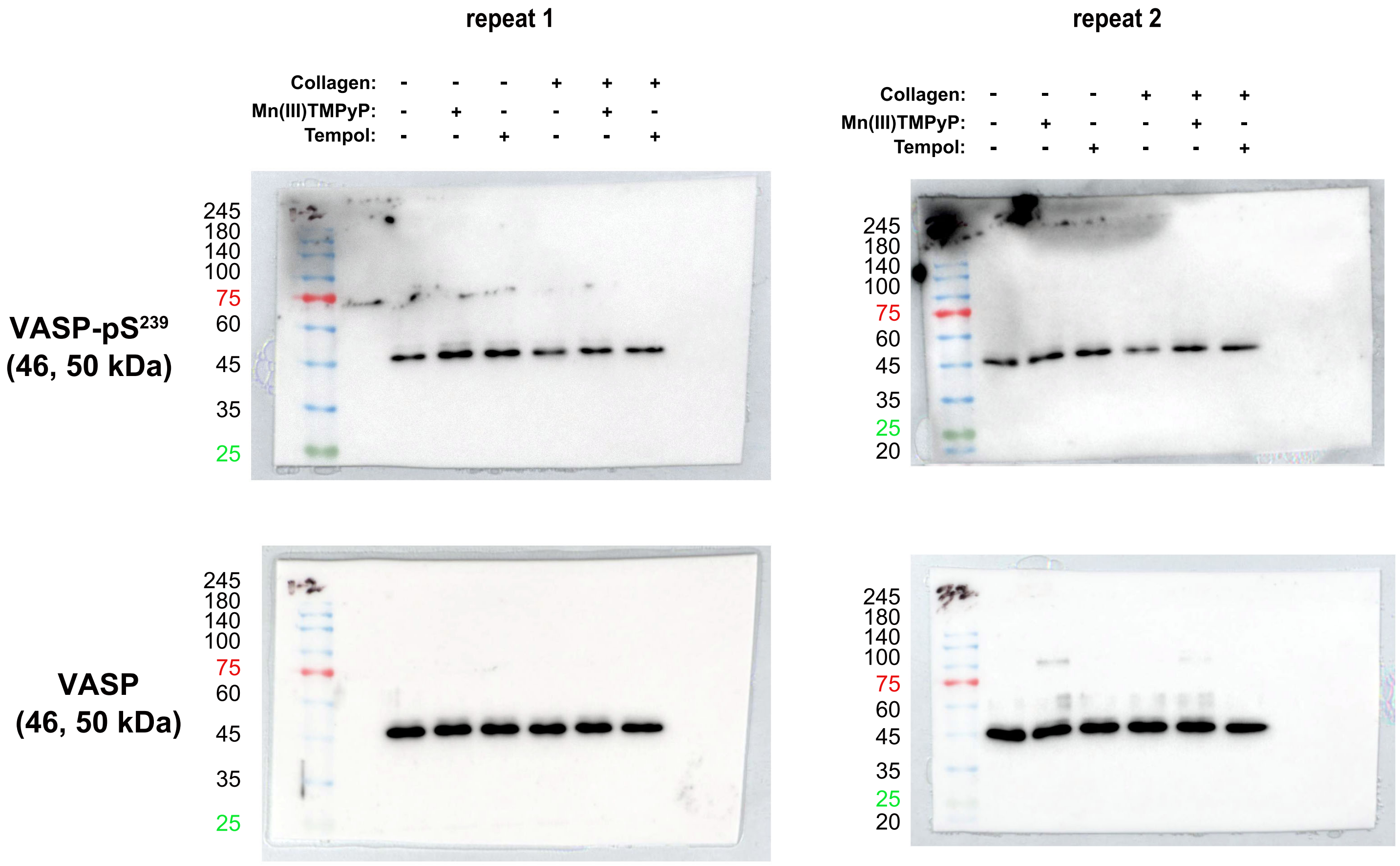

Full uncropped immunoblots of Supplementary Figure S4C.
The repeat 1 is used in Supplementary Figure S4C.

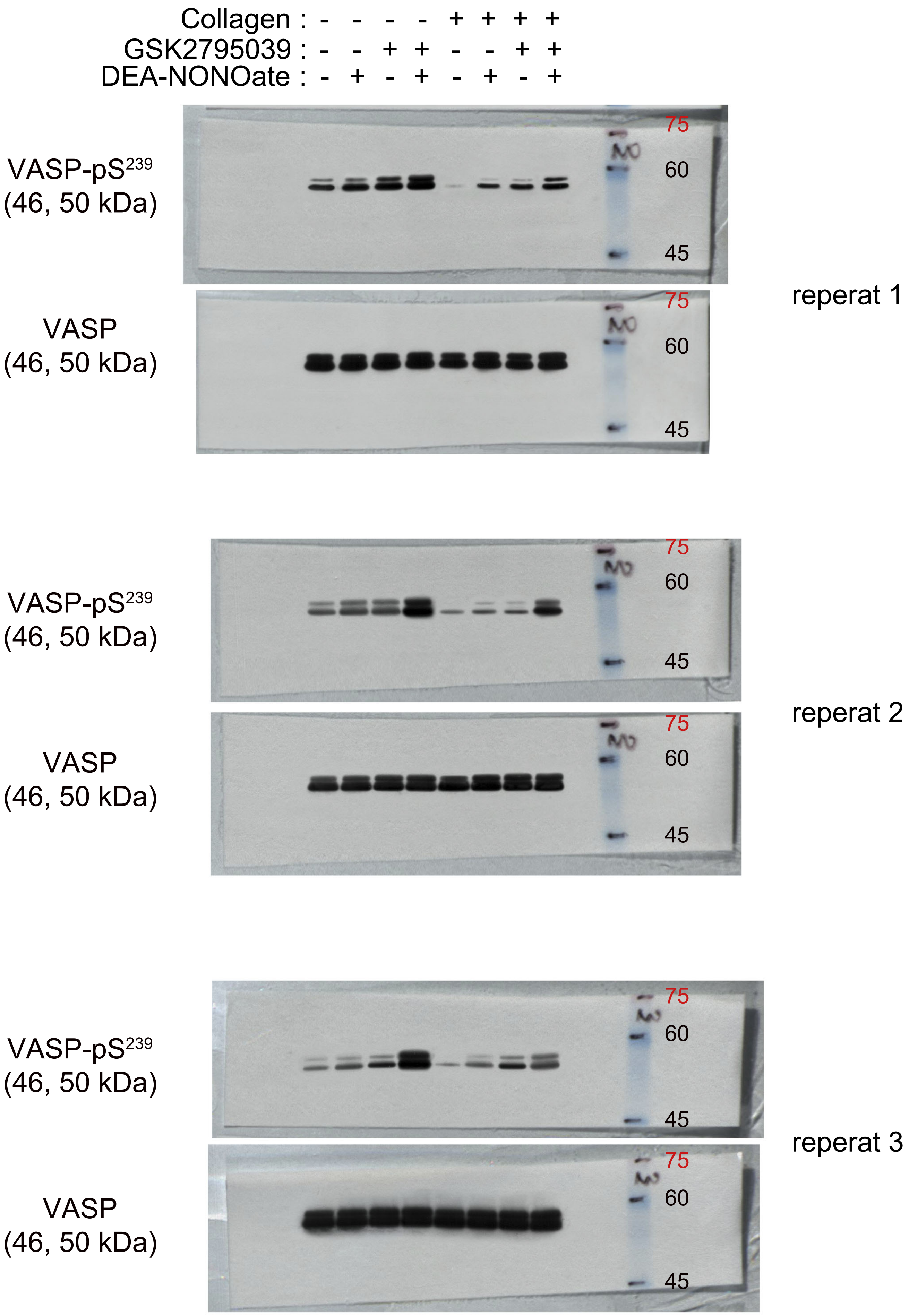

Full uncropped immunoblots of Supplementary Figure S4D.
The repeat 1 is used in Supplementary Figure S4D.

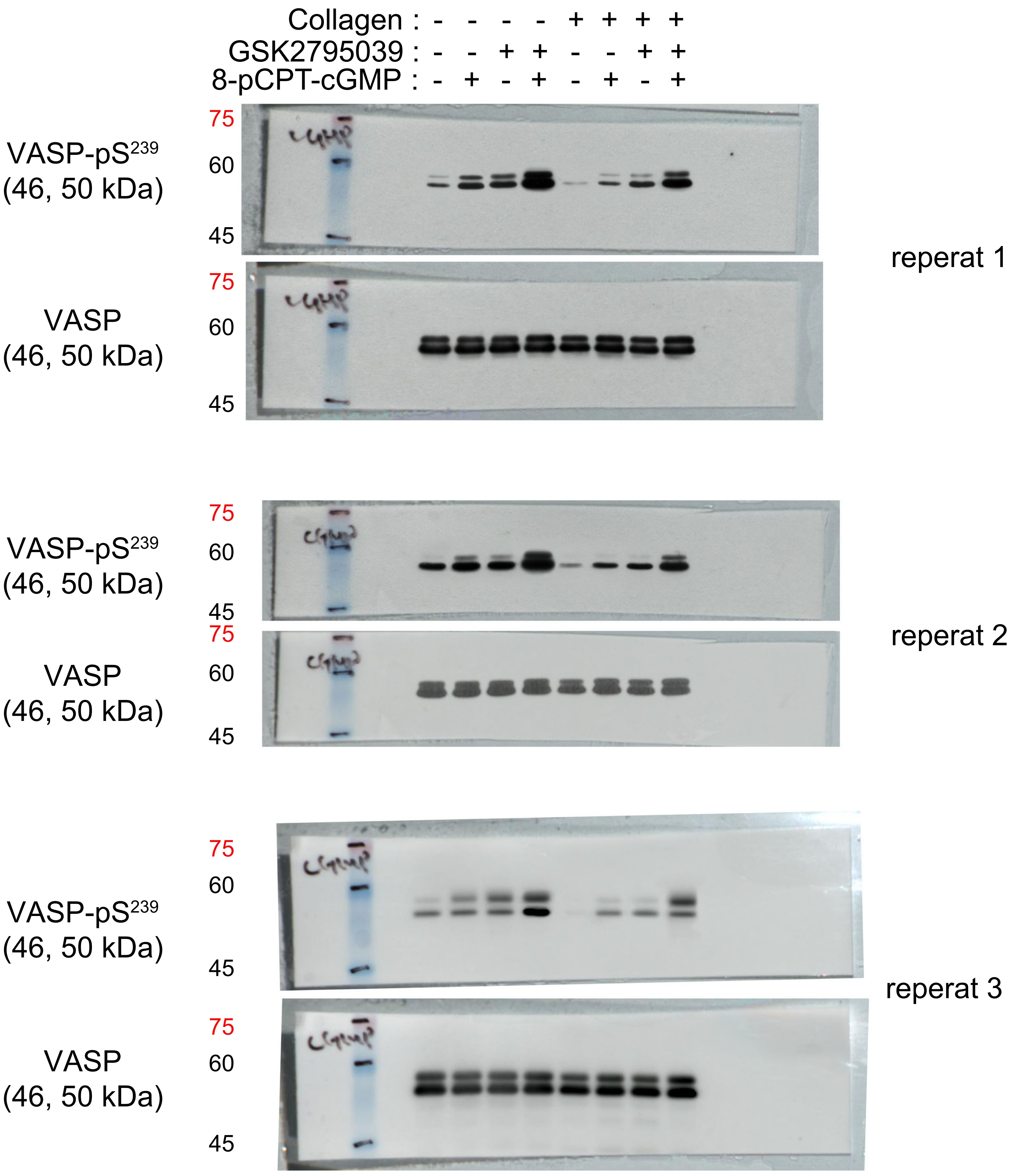

Supplement: Supplementary file 1 — Supplementary Material 1 [file 41598_2025_20250_MOESM1_ESM.pdf]
